# The Effects of Cannabidiol, a Non-Intoxicating Compound of Cannabis, on the Cardiovascular System in Health and Disease

**DOI:** 10.3390/ijms21186740

**Published:** 2020-09-14

**Authors:** Aleksandra Kicman, Marek Toczek

**Affiliations:** Department of Experimental Physiology and Pathophysiology, Medical University of Białystok, 15-222 Białystok, Poland; olakicman@gmail.com

**Keywords:** cannabidiol, cannabinoids, cannabis, cardiovascular system, hypertension, ischemia, cardiomyopathy, vasodilation

## Abstract

Cannabidiol (CBD) is a non-intoxicating and generally well-tolerated constituent of cannabis which exhibits potential beneficial properties in a wide range of diseases, including cardiovascular disorders. Due to its complex mechanism of action, CBD may affect the cardiovascular system in different ways. Thus, we reviewed the influence of CBD on this system in health and disease to determine the potential risk of cardiovascular side effects during CBD use for medical and wellness purposes and to elucidate its therapeutic potential in cardiovascular diseases. Administration of CBD to healthy volunteers or animals usually does not markedly affect hemodynamic parameters. Although CBD has been found to exhibit vasodilatory and antioxidant properties in hypertension, it has not affected blood pressure in hypertensive animals. Hypotensive action of CBD has been mainly revealed under stress conditions. Many positive effects of CBD have been observed in experimental models of heart diseases (myocardial infarction, cardiomyopathy, myocarditis), stroke, neonatal hypoxic ischemic encephalopathy, sepsis-related encephalitis, cardiovascular complications of diabetes, and ischemia/reperfusion injures of liver and kidneys. In these pathological conditions CBD decreased organ damage and dysfunction, oxidative and nitrative stress, inflammatory processes and apoptosis, among others. Nevertheless, further clinical research is needed to recommend the use of CBD in the treatment of cardiovascular diseases.

## 1. Introduction

*Cannabis sativa* has been used since ancient times for agricultural, ceremonial and medicinal purposes. In traditional medicine, the plant has been used as an analgesic, anticonvulsant, anti-asthmatic, antimalarial or anti-rheumatic agent. Cannabis contains over 700 different chemicals, among which a group of compounds called cannabinoids stands out. Cannabinoids found in cannabis are called phytocannabinoids. Beyond the plant-derived cannabinoids, there are also cannabinoids endogenously produced in humans or animals (so-called endocannabinoids) and synthetic cannabinoids [1,2]. More than 100 phytocannabinoids have been identified, and the two best known of them (for a comparison, see Table 1) are Δ^9^-tetrahydrocannabinol (THC, more precisely its isomer (-)-trans) and cannabidiol (CBD). THC is the main psychoactive ingredient in cannabis and, due to its intoxicating effects, marijuana, hashish or hash oil are commonly-used illicit drugs. In contrast, CBD is generally considered to be a non-intoxicating substance (it is often described in the literature as ‘non-psychoactive’, however, it may modulate symptoms of certain neuropsychiatric disorders; therefore, the term ‘non-intoxicating’ tends to be preferable), neither causing addiction nor inducing serious side effects [1,3,4,5]. In addition, it can modulate THC actions and, therefore, reduce or potentiate (dependently on the dose and CBD:THC ratio) side effects of THC [6,7]. 

Basic and/or clinical studies have shown that cannabidiol has multidirectional properties (Table 1), such as antioxidant, anti-inflammatory, immunomodulatory, antiarthritic, anticonvulsant, neuroprotective, procognitive, anti-anxiety, antipsychotic and anti-proliferative, among others. Thus, CBD possesses wide therapeutic potential, which includes e.g., epilepsy, neurodegenerative diseases (multiple sclerosis, Alzheimer’s, Parkinson’s and Huntington’s diseases), neuropsychiatric disorders (depression, anxiety disorders, schizophrenia, post-traumatic stress disorder, autistic spectrum disorders), gastrointestinal disorders (nausea and vomiting, inflammatory bowel diseases, irritable bowel syndrome), rheumatic diseases, graft versus host disease and cancer (reviewed elsewhere: [5,6,17,30,31,32,33]). However, most of these indications require further investigation to confirm clinical effectiveness.

The first drug to exclusively use cannabidiol as its active ingredient was registered in June 2018 in the USA under the name Epidiolex^®^ (GW Pharmaceuticals, UK). It is a liquid preparation containing CBD (100 mg/mL) of plant origin and indicated for use in severe drug-resistant epilepsy manifest during early childhood such as Dravet syndrome and Lennox-Gastaut syndrome [28]. In the European Union, CBD currently has only orphan drug status in several diseases, such as epileptic syndromes (listed above and West syndrome), perinatal asphyxia, tuberous sclerosis, graft versus host disease and glioma (combined with THC in the latter one) [34]. In turn, in many European countries nabiximols (trade name Sativex^®^, GW Pharmaceuticals), a cannabis extract containing CBD and THC at an approximate 1:1 ratio, is available. Sativex^®^ is administered as an oral spray and was developed to relieve the symptoms of spasticity in patients with multiple sclerosis [4]. In addition, CBD and THC are present in varying proportions in marijuana and derivatives have been developed for medical applications (so-called medical marijuana or medical cannabis) [4,29]. It should be mentioned that CBD is also found in dietary supplements, creams and lotions for topical administration and oils for vaporization [30,33]. Increased interest in the health-promoting and therapeutic properties of these products has led to widespread use, which can be associated with potential adverse effects or interactions with co-administered drugs. 

The endocannabinoid system comprised of endocannabinoids, their synthesizing and metabolizing enzymes and cannabinoid receptors (CB_1_ and CB_2_) is present in the cardiovascular system. Both endogenous and exogenous cannabinoids induce changes in the cardiovascular system of humans and animals [19,20,35]. Cardiovascular complications, such as tachycardia and acute coronary events, are associated widely with marijuana smoking (the effects mainly dependent on THC) or intake of synthetic cannabimimetics as a constituent in designer drugs [36]. In turn, CBD is devoid of adverse cardiovascular effects. In addition, it has been suggested to have therapeutic potential in the treatment of the cardiovascular diseases such as stroke, myocardial infarction, myocarditis, cardiomyopathies and cardiovascular complications of diabetes, which is connected with vasodilatory, cardioprotective, antioxidant, anti-inflammatory and neuroprotective properties of CBD [23,24].

## 2. Biosynthesis and Pharmacology of Cannabidiol

### 2.1. Structure and Biosynthesis

Cannabidiol (see chemical structure, Table 1), like other cannabinoids, belongs to group C21 (or C22 for carboxylated forms) of terpenophenols. In its acidic form (see below), cannabidiol is the main components of cannabis fibrous varieties [1,4]. It was first isolated from cannabis by Adams et al. in the UK [37] and from hashish by Jacob and Todd in the USA [38] in 1940. However, its chemical structure was not determined until 1963 by Israeli scientists Mechoulam and Schvo [39], and its absolute configuration four years later by Mechoulam and Gaoni [40]. 

The biosynthesis and storage of cannabidiol, and other phytocannabinoids, occurs in the glandular trichomes present mainly on female flowers. Lower amounts of phytocannabinoids are also detected in leaves, stems, seeds, roots or pollen. Aside from the type of tissue, the concentrations of bioactive compounds in cannabis depend on variety, growth conditions, growing stage, harvest time and conditions of storage [3,41].

**Figure 1:**
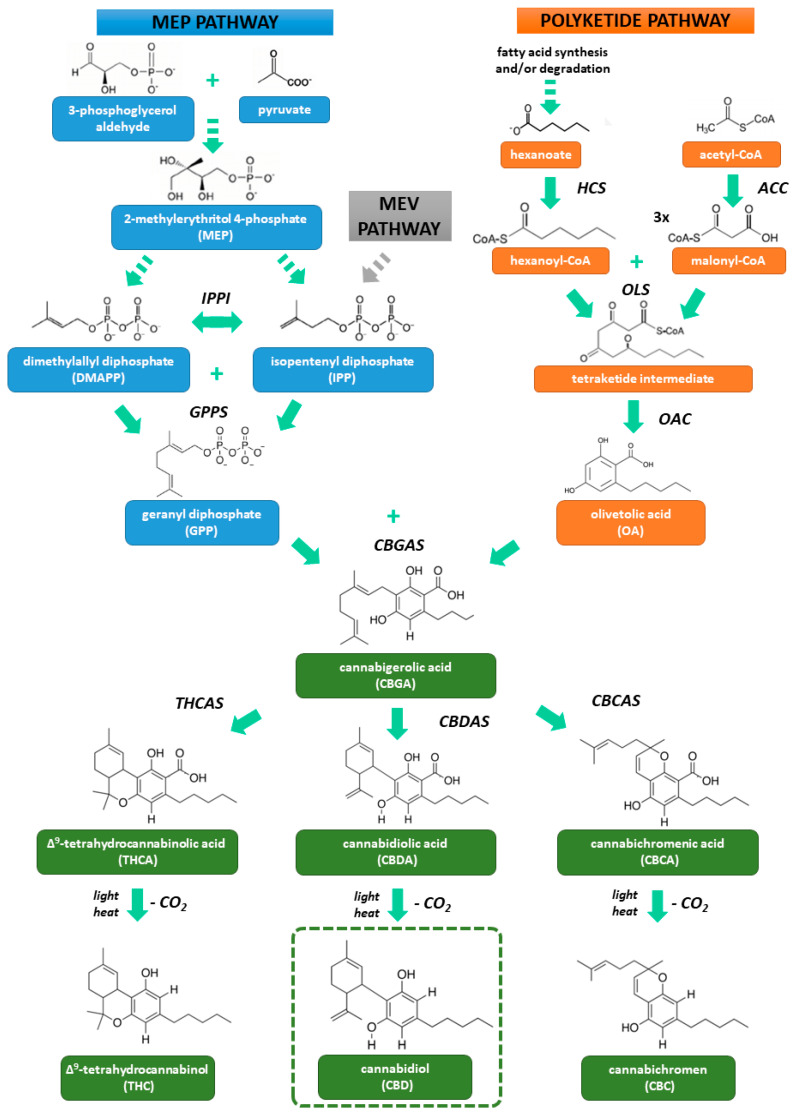
Biosynthesis of cannabidiol and other phytocannabinoids [1,42,43]. Abbreviations: ACC: acetyl-CoA carboxylase; CBCAS: cannabichromenic acid synthase; CBDAS: cannabidiolic acid and its synthase; CBGAS: cannabigerolic acid synthase; GPPS: geranyl diphosphate synthase; HCS: hexanoyl-CoA synthetase; IPP: isopentenyl diphosphate; MEV: mevalonic acid; OAC: olivetoleic acid cyclase; OLS: olivetol synthase; THCAS: Δ^9^-tetrahydrocannabinolic acid synthase.

The biosynthesis of phytocannnabinoids (Figure 1) starts with the synthesis of two precursor compounds—geranyl diphosphate (GPP) in the 2-methylerythritol 4-phosphate (MEP) pathway and olivetoleic acid (OA) in the polyketide pathway. The terpene moiety of cannabinoids comes from GPP which is formed through condensation of isopentenyl diphosphate (IPP) and dimethylallyl diphosphate (DMAPP) catalysed by GPP synthase [1,42]. IPP and DMAPP are isomeric (they undergo mutual transformation under the influence of IPP isomerase), and are synthesized in plastids via the MEP pathway. Pyruvate and 3-phosphoglycerol aldehyde are MEP precursors. The cytosolic mevalonic acid (MEV) pathway can also be a source of IPP and DMAPP, but for cannabinoid synthesis this is likely to be minor [42,43]. The second important precursor of cannabinoids is OA responsible for their phenolic moiety. It is formed as a result of aldol condensation of hexanoyl-CoA with three molecules of malonyl-CoA, and for this transformation two enzymes are necessary—olivetol synthase and olivetolic acid cyclase acting on the resulting tetraketide intermediate product. Hexanoyl-CoA is the product of a reaction between hexanoate (formed by biosynthesis and/or degradation of fatty acids) and CoA, catalysed by synthetase of hexanoyl-CoA. Malonyl-CoA, on the other hand, is a product of acetyl-CoA carboxylation by acetyl-CoA carboxylase [42]. 

Fusion of OA and GPP produces cannabigerolic acid (CBGA) catalysed by its synthase. CBGA is considered to be the central precursor compound for phytocannabinoids. Cannabidiolic acid synthase converts CBGA into cannabidiolic acid (CBDA). Similarly, tetrahydrocannabinolic acid (THCA) and cannabichromenic acid (CBCA) are formed by their specific synthases. Acidic types of cannabinoids are susceptible to light and heat and as a result of non-enzymatic decarboxylation they are converted to neutral forms—CBD, THC and cannabichromene (CBC) [1,42]. It should be noted that neutral forms occur in the growing plant at low concentrations, and only during the heat treatment of the raw material (burning, baking) are they formed in large quantities [44]. 

### 2.2. Mechanism of Action

Cannabinoids exert their effects via interaction with the cannabinoid receptors CB_1_ and CB_2_, discovered in the early 1990s. They are metabotropic receptors associated with G_i/o_ proteins and their stimulation results in inhibition of adenylyl cyclase and stimulation of mitogen-activated protein kinases (MAPK) and (only for CB_1_) modulation of calcium and potassium channels. Moreover, the effects of CB_1_ receptors can be mediated by G_q_ and G_s_ proteins and independently of G proteins. CB_1_ receptors are located mainly in the central nervous system, while the CB_2_ receptors are found in abundance in the immune system. Thus, cannabinoids can exert important pro-homeostatic physiological functions by modulating neurotransmitter release and immune responses. It is worth noting that the presence of both types of receptors has been demonstrated throughout the body and their expression may change in pathological conditions [17,22,27]. 

Cannabidiol has low affinity for cannabinoid receptors (at micromole concentrations) [22]. It does not induce effects typical for stimulation of central CB_1_ receptors, such as hypoalgesia, hypothermia, catalepsy and decreased motor activity (the so-called cannabinoid tetrad), which are characteristic for THC [18]. Moreover, CBD is capable of antagonizing the actions of CB_1_/CB_2_ receptor agonists (CP55940 and R-(+)-WIN55212) at nanomole concentrations, therefore, lower than those resulting from its affinity to these receptors [26]. It has been demonstrated that CBD is an inverse agonist of CB_2_ receptor [26] and a negative allosteric modulator of CB_1_ receptors [12]. Despite the lack of agonistic properties of CB_1_/CB_2_ receptors by CBD, some of its effects are inhibited by antagonists/inverse agonists of these receptors [8,45] or are not present in CB_1_ knockout mice [46]. This is most likely an effect of indirect cannabinomimetics action of CBD as its administration has been shown to increase the concentration of endogenous cannabinoids—anandamide (AEA) [8,14,15] and 2-arachidonoylglycerol (2-AG) [45]. The mechanisms of this effect may include decreased breakdown and intracellular endocannabinoid uptake. CBD inhibits the major enzyme responsible for breakdown of AEA (and to a lesser extent 2-AG) [27]—fatty acid amide hydrolase (FAAH), in rodents [9,16], but not in humans [10]. Moreover, CBD inhibits AEA uptake by acting on a putative endocannabinoid membrane transporter (EMT) [9,16] and/or competition with AEA for binding to fatty acid binding proteins (FABP-3, -5, -7), which constitute an intracellular endocannabinoid transport system [10].

Multiple studies have shown that CBD has many effects independent of direct or indirect interaction with CB_1_/CB_2_ receptors. The agonistic action of the CBD has been demonstrated at the following: transient receptor potential ankyrin subfamily member 1 (TRPA1) and vanilloid subfamily members 1–4 (TRPV1–4), peroxisome proliferator-activated receptor γ (PPARγ), orphan G-protein coupled receptor—GPR18 (CBD is a partial agonist but antagonizes THC effects) and serotonin 5-HT_1A_ and 5-HT_2A_ receptors (partial agonist). Moreover, CBD is a positive allosteric modulator of α1-, α1β- and α3-glycine receptors (α1-, α1β- and α3-GlyR), μ- and δ-opioid receptors (μ- and δ-OR) and γ-aminobutyric acid receptor type A (GABA_A_). In contrast, CBD shows antagonistic activity at the orphan receptor GPR55 (even postulated as CB_3_ receptor), the putative receptor for abnormal-cannabidiol (Abn-CBD; see below) and the transient receptor potential melastatin subfamily member 8 (TRPM8). Moreover, it is a negative allosteric modulator of serotonin 5-HT_3_ receptor, α_1_-adrenergic receptor (α_1_-AR) and dopamine D_2_ receptor [11,17,21]. Recently it has also been shown that CBD is an inverse agonist for orphan receptors GPR3, GPR6 and GPR12 [13]. 

In addition to direct exposure to a number of receptors, CBD can also exert its effects by indirectly increasing the concentration of biologically active compounds. Aside from the above-mentioned effect on endocannabinoid levels, CBD inhibits, e.g., adenosine, thymidine, glutamate, serotonin, γ-aminobutyric acid, dopamine and noradrenaline uptake. The level of serotonin may also be modulated by inhibiting the decomposition of its precursor, tryptophan. Additionally, CBD also affects the metabolism of arachidonic acid by affecting phospholipase A_2_ (PLA_2_; stimulation or inhibition depending on the CBD concentration), 5- and 15-lipooxygenase (5-, 15-LOX; inhibition) or cyclooxygenase isoenzymes (COX-1 and -2; inhibition or stimulation) activity. In consequence, both decreased and increased prostaglandin E (PGE) production have been demonstrated [11,17,21]. Considering the close association of arachidonic acid and endocannabinoid metabolic pathways (common metabolic enzymes; arachidonic acid is formed from endocannabinoid decomposition) [27], CBD can comprehensively affect the formation of a large group of mediators—arachidonic acid derivatives and endocannabinoids.

**Figure 2:**
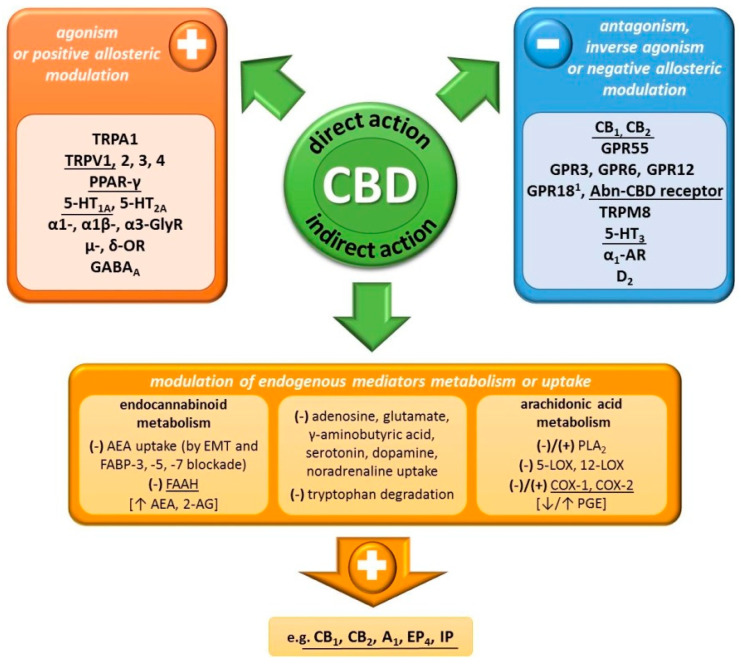
Cannabidiol (CBD) mechanism of action [8,9,10,11,12,13,14,15,16,17,21,22,26,27,45,46]. The mechanisms through which CBD exerts its effects in the cardiovascular system are underlined [23,30,47,48,49,50,51,52,53,54,55,56,57]. ^1^CBD is a low efficacy partial agonist of GPR18 and antagonizes THC effects (CBD acts as an antagonist); abbreviations: 5-, 12-LOX: 5-,12-Lipoxygenase; 2-AG: 2-Arachidonoylglycerol; 5-HT_1A, 2A, 3_: Serotonin receptors type 1A, 2A, 3; Abn-CBD: Abnormal-cannabidiol; AEA: Anandamide; A_1_: Adenosine receptor type 1; CB_1, 2_: Cannabinoid receptor type 1, 2; COX-1,-2: Cyclooxygenase 1, 2; D_2_: Dopamine receptor type 2; EMT: Endocannabinoid membrane transporter; EP_4_: Prostaglandin E receptor 4; FAAH: Fatty acid amide hydrolase; FABP-3,-5,-7: Fatty acid binding protein 3, 5, 7; GABA_A_: γ-Aminobutyric acid receptor type A; GPR3, 6, 12, 18, 55: G-protein coupled receptor 3, 6, 12, 18, 55; IP: Prostacyclin receptor; PGE: Prostaglandin E; PPAR-γ: Peroxisome proliferator-activated receptor γ; TRPA1: Transient receptor potential ankyrin subfamily member 1; TRPM8: Transient receptor potential melastatin subfamily member 8; TRPV1-4: Transient receptor potential vanilloid subfamily members 1-4; α1-, α1β-, α3-GlyR: α1, α1β-, α3-Glycine receptor; α_1_-AR: α_1_-Adrenergic receptor; δ-, μ-OR: δ-, μ-Opioid receptor.

In summary, CBD has a complex pharmacodynamic profile (Figure 2). However, in many cases its activity occurs at very high concentrations and to date only in vitro. Nevertheless, such a complex mechanism of action might explain wide therapeutical potential of CBD.

### 2.3. Pharmacokinetics

There are different routes of cannabidiol administration, of which the inhalation (smoking, vaporization or nebulization) and oral (oils, capsules, food and drinks enriched with CBD) routes are the most common. In therapeutic applications, it can also be administered as an oromucosal spray (Sativex*^®^*). It can also be administered intravenously, percutaneously, rectally or in the form of eye drops [4,30,33]. The bioavailability varies depending on the route of administration, e.g., for the inhalation route it is estimated at 31% and maximum concentrations are reached 3–10 min after consumption. For the oral route, on the other hand, maximum concentrations are reached after 1–2 or up to six hours after intake, and bioavailability is less than 20%, due to the first-pass metabolism [4,58].

**Figure 3:**
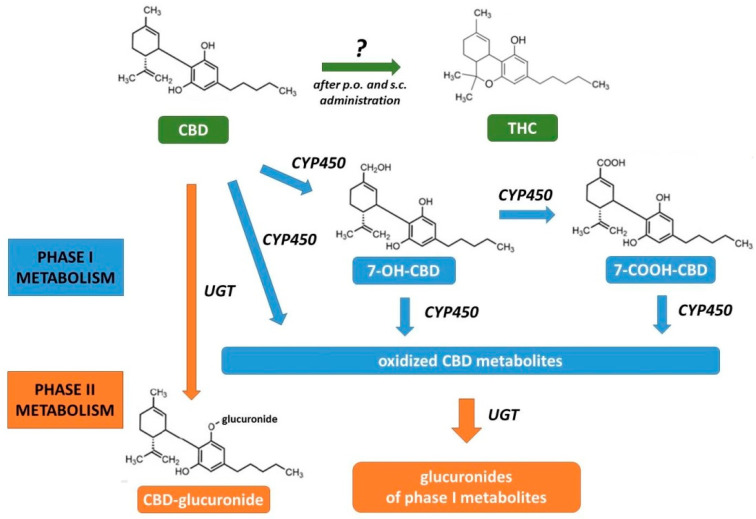
Metabolism of cannabidiol [59,60,61,62,63,64,65,67,68]. Abbreviations: 7-COOH-, 7-OH-CBD:7-Carboxy-, 7-hydroxycannabidiol; CBD: Cannabidiol; CYP: Cytochrome P450; p.o.: Per os, orally; s.c.: Subcutaneously; THC: Δ^9^-Tetrahydrocannabinol; UGT: UDP-Glucuronosyltransferase.

The oral route has been associated with possible CBD transformation to THC in the acidic gastric environment suggested by some authors (Figure 3). Such conversion was found in a studies with simulated gastric fluid [59,60]. However, it seems that this conversion does not occur in vivo in humans, as evidenced by the absence of THC in the blood of patients who took even very high doses of CBD orally. Moreover, this compound does not cause any psychological, psychomotor, cognitive or physiological effects typical for THC or marijuana. This discrepancy may be explained by the fact that gastric juices do not perfectly mimic the real conditions in the stomach [61,62]. Studies on animals also show conflicting data. Hložek et al. [63] demonstrated the presence of THC in rat blood after oral (and also subcutaneous) administration of CBD. However, Palazzoli et al. [64] reported no THC in the blood of rats orally administered a single high dose of CBD both three and six hours after administration. Similarly, THC was not found in guinea pigs receiving oral CBD for five days [65]. Thus, possible CBD conversion to THC seems doubtful. 

Cannabidiol is transported in the blood mainly in protein-bound form and about 10% of CBD binds to erythrocytes. It is rapidly distributed to all organs well supplied with blood, such as the brain, heart, lungs and liver. The distribution volume of CBD is about 32 L/kg. Due to its high lipophilicity, it may accumulate in adipose tissue when used chronically [4,66]. 

Cannabidiol is eliminated through metabolism and excretion. CBD is excreted both in the unaltered state and in the form of metabolites with urine and faeces [4,67]. The reported half-life of CBD in humans depends on the study (different doses, routes of administration) and may vary from about one hour to five days [58,67]. Cannabidiol undergoes biotransformation consisting of two phases (Figure 3). The first occurs mainly in the liver, where CBD undergoes transformations involving isoenzymes of cytochrome P450 (CYP). In a study with human recombinant CYP it has been shown that CYP1A1, CYP1A2, CYP2C9, CYP2C19, CYP2D6, CYP3A4 and CYP3A5 can metabolize CBD, of which CYP3A4 and CYP2C19 play a dominant role in liver microsomes [68]. The metabolic profiles of CBD vary according to species. About 40 different phase I metabolites have been identified in humans and the main ones are 7-carboxy-cannabidiol (7-COOH-CBD) derivatives. The pharmacological activity of phase I metabolites is different, e.g., 7-hydroxy-cannabidiol (7-OH-CBD) like CBD inhibits FAAH and AEA uptake, whereas 7-COOH-CBD does not show such activity; both 7-OH-CBD and 7-COOH-CBD are not TRPV1 agonists. Both CBD and its oxidized phase I metabolites undergo glucuronidation which is the main reaction of phase II [67].

It is worth noting that CBD is not only a substrate for CYP isoenzymes, but may also affect their activity. It has been shown that CBD is an inhibitor of CYP1A1, CYP1A2, CYP1B1, CYP2C19, CYP2C9, CYP2D6, CYP3A4, CYP3A5 and CYP3A7 [11]. On the other hand, longer CBD intake may induce expression of some isoenzymes, as demonstrated in mice for CYP3A and CYP2B10. CBD also induced expression of CYP1A1 in human liver cells. Thus, the influence on the activity/expression of CYP isoenzymes results in the possibility of CBD interaction with other concomitantly used drugs [67,69]. In the area of cardiovascular drugs one case of such interaction has been described so far. Concomitant use of warfarin (anticoagulant) and CBD (Epidiolex^®^) intensified the antithrombotic effect (increase of the international normalized ratio, INR). As suggested by the authors, this may have resulted from competition for binding and inhibition by CBD of CYP isoenzymes involved in warfarin metabolism [70]. Therefore, caution should be exercised while simultaneously taking CBD with other drugs. 

## 3. Effects of Cannabidiol on the Cardiovascular System under Physiological Conditions 

The administration of phyto-, endo- and synthetic cannabinoids has a diverse and sometimes polyphasic influence on blood pressure (BP) and heart rate (HR) depending on the species, route of administration, presence of anaesthesia and other experimental conditions [19]. Cannabinoids can affect cardiovascular function not only by cannabinoid receptors, but a variety of other receptors, located both in the nervous system and directly in the blood vessels and heart [19,35,71,72]. Stimulation of central CB_1_ receptors causes an increase in blood pressure, whereas peripheral CB_1_ receptors located presynaptically at the endings of pre- and/or postganglionic sympathetic neurons innervating the heart and vascular resistance are responsible for hypotensive effects of cannabinoids. Activation of CB_1_ receptors located in the myocardium reduces contractility. In addition, cannabinoids can stimulate or inhibit the Bezold-Jarisch reflex, characterized by short and strong bradycardia and hypotension, via TRPV1 and 5-HT_3_ receptors located on sensory vagal nerve fibres [19,72]. Cannabinoids, in most cases, cause vasodilation in isolated blood vessels or perfused vascular beds, although vasoconstriction is also observed. These effects result from direct activation of CB_1_, TRPV1, PPARs and the putative endothelial cannabinoid receptors. In addition, the role of CB_2_, GPR55 and 5-HT_1A_ receptors in vasoactive effects of cannabinoids has been revealed in some studies [71,72]. Cannabinoids can also activate orphan GPR18 localized peripherally in blood vessels (GPR18 was deorphanised as the endothelial cannabinoid receptor by some authors, see below) and centrally in rostral ventrolateral medulla (RVLM) which results in vasorelaxation and hypotension [35,73]. Cannabinoids may also affect the cardiovascular system through their metabolites, e.g., prostanoids. Thus, vasorelaxation or vasoconstriction evoked by cannabinoids was mediated indirectly through prostaglandin E receptor 4 (EP_4_) and prostacyclin receptor (IP) or prostaglandin E receptor 1 (EP_1_) and thromboxane receptor (TP), respectively [19,71]. 

The search for relevant studies investigating the effect of CBD on cardiovascular system under physiological conditions was performed via electronic searches of three databases (PubMed, Cochrane Library and EBSCO) from their inception to March 2020. Search keywords included: cannabidiol and cardiovascular, haemodynamic, blood pressure, heart rate, blood flow, blood vessels, heart, vasodilation or vasorelaxant. References from included studies were also hand searched. 

The complex mechanism of action of cannabidiol makes it possible to have multidirectional influence on the cardiovascular system. However, studies carried out, to date, in animals and humans largely indicate no or little effect of CBD administered orally (p.o), intravenously (i.v), intra-arterially, intraperitoneally (i.p.), centrally or through inhalation (after acute and repeated dosing) on systolic (SBP), diastolic (DBP) or mean (MBP) arterial blood pressure and/or heart rate under physiological conditions (Table 2) [7,15,49,55,74,75,76,77,78,79,80,81,82,83,84,85,86,87,88,89,90,91,92,93,94,95,96,97]. This is confirmed by the results of a meta-analysis by Sultan et al. [24], which indicated no influence of CBD on HR and BP after acute and chronic administration (in the latter case only HR was analysed). However, there are some exceptions. In humans, CBD slightly increased (at dose of 40 mg, but not 20 mg, given sublingually) [98] or decreased resting BP (600 mg, p.o.) after acute dosing [97,99], but not after repeated dosing (600 mg for 7 days, p.o.) [97]. Conversely, no tolerance for hypotensive effect of CBD was observed after its chronic oral dosing rising from 100 to 600 mg/day over 6 weeks in patients with dystonic movement disorders [100]. It has also been shown that CBD can increase regional cerebral blood flow (CBF) [101]. The influence of CBD on cardiovascular system in humans might depend not only on a dose [98] and duration of administration [97], but also on the delivery method of CBD. Thus, oral CBD at dose of 90 mg did not influence BP, HR and CBF, however the same dose of CBD encapsulated as TurboCBD^TM^ (patented capsule formulation increasing CBD bioavailability) decreased DBP and MBP and increased CBF [94]. In animals, CBD can also affect cardiovascular parameters variously. It increased MBP and HR in pentobarbital anaesthetised dogs [102], decreased HR in conscious rabbits [103], reduced MBP in mice anaesthetised with ketamine and xylazine [104], slightly raised SBP, DBP and HR in conscious rats [52] and decreased MBP in pentobarbital anaesthetised rats [104,105]. In urethane anesthetised rats, CBD administered intravenously did not influence hemodynamic parameters [74]. However, when injected quickly, it can induce the Bezold-Jarisch reflex via TRPV1 receptors. Additionally, CBD diminished the Bezold-Jarisch reflex induced by 5-HT_3_ receptor activation [52]. CBD can also modify the baroreflex response after central (into the bed nucleus of the stria terminalis, BNST) administration. Thus, it exhibited a facilitatory influence on the reflex bradycardiac response to blood pressure increases via 5-HT_1A_ receptors activation [47]. After pithing and vagotomy in rats anaesthetised with urethane, i.e., after abolition of reflex responses and the influence of central nervous system on the cardiovascular system, CBD-induced an increase in HR and systolic pressure, whereas a decrease in diastolic pressure probably resulted from vasodilation [52]. 

The vasodilatory effect of CBD has been demonstrated on isolated human and animal vessels under both physiological (Table 3) [48,56,106] and pathological (see below) conditions and it is probably the most consistent effect of this compound in the cardiovascular system. The mechanism of CBD action on vessels is complex and depends on the examined vascular bed, however, it does not include cannabinoid receptors [48,106], except for isolated human mesenteric arteries, where dependence on CB_1_ receptors has been demonstrated [56]. It is worth noting that CBD causes time-dependent vasodilation through nuclear receptors PPAR-γ [48,56,106]. Nevertheless, the vasodilatory action of CBD ex vivo, in most cases, are not translated into systemic blood pressure (no decrease in BP after CBD administration, see above). Experiments on pithed and vagotomised rats where CBD decreased DBP have indicated that vasorelaxant action of CBD in vivo can be masked by neurogenic tone [52]. Studies on human aortic endothelial cells (Table 3) showed that CBD reduced phosphorylation of c-Jun N-terminal kinase (JNK), *nuclear factor* κB (NF-κB), ribosomal protein S6 kinase (p70S6K) and *signal transducer and activator of transcription 5* (STAT5), and increased phosphorylation of *cAMP response element*-*binding protein* (CREB), *extracellular signal*-*regulated kinase 1/2 (*ERK1/2), protein kinase B (Akt) and *endothelial* nitric oxide synthase (NOS). These alterations in the phosphorylation of the intracellular proteins might explain the vasodilatory (ERK, Akt and endothelial NOS), anti-angiogenic (p70S6K and STAT5) and anti-inflammatory (JNK and NF-κB) properties of CBD [56]. Cannabidiol also exerts positive effects on vascular smooth muscle cells (Table 3) which aberrant proliferation and migration are linked to the development and progression of cancer and cardiovascular diseases. Thus, CBD inhibited proliferation and migration of human umbilical artery smooth muscle cells and increased cytoprotective enzyme heme oxygenase-1. The latter effect was accompanied by CBD-induced reactive oxygen species production [107]. This is surprising observation given the many studies demonstrating antioxidant properties of CBD [31]. However, in another study, CBD given chronically decreased oxidative stress markers in rats with hypertension, but exhibited prooxidative effects in normotensive control animals, especially in Wistar–Kyoto rats (Table 2) [96]. 

Studies on isolated hearts, atria or single cardiomyocytes (Table 3) indicate a direct negative inotropic effect of cannabidiol [52,108,109]. In isolated hearts, CBD may decrease [108] or slightly increase [110] heart rate and may also have a proarrhythmic effect [108]. Studies in pithed and vagotomised rats have shown that CBD can indirectly affect the heart by exerting a peripheral sympathomimetic effect (manifested by an increase in HR and SBP), presumably due to the effect on the release and/or re-uptake of noradrenaline from sympathetic terminals [52]. An increase in heart rate was also demonstrated in some human and animal studies (Table 2) [52,99,102]. Additionally, in the meta-analysis by Sultan et al. [24], the rat subgroup showed an increase in HR after CBD administration. However, in most cases, CBD does not affect HR in vivo, which indicates (similarly to blood vessels) that peripheral effects might be masked by central influences. 

**Table 3:** In vitro and ex vivo effects of cannabidiol (CBD) in cardiovascular system under physiological ^1^ conditions.

Species	Organ/Cells	Concentration	Effects ^2^	References
Human ^3^	Isolated mesenteric arteries (pre-constricted with U46619 ^4^ and endothelin-1)	0.1–100 μmol/L	- Vasodilation (effect is dependent on CB_1_, TRP, endothelium and NO; independent on CB_2_, Abn-CBD receptor, COX, and potassium channels)	[56]
		10 μmol/L (time-dependent effect)	- Vasodilation (effect is independent on PPAR-γ)	
Human ^5^	Isolated pulmonary arteries (pre-constricted with U46619 ^4^)	0.1–30 μmol/L	- Vasodilation (effect is dependent on endothelium, COX, EP_4_, IP, TRPV1; independent on CB_1_ and CB_2_)	[48]
		10 μmol/L (time-dependent effect)	- Vasodilation (effect is dependent on PPAR-γ)	
Human	Human aortic endothelialcells (HAEC)	0.1–30 μmol/L	- ↓ phosphorylation of JNK, NF-κB, p70S6K and STAT5; - ↑ phosphorylation of CREB, ERK1/2 (effect is dependent on CB_1_ and TRPV_1_), Akt (effect is dependent on CB_1_) and endothelial NOS (effect is dependent on CB_1_)	[56]
Human	Human umbilical artery smoothmuscle cells (HUASMC)	0.1–10 μmol/L	- ↑ expression of HO-1 (effect is dependent on ROS; independent on CB_1_, CB_2_, GPR55, TRPV1); - ↓ migration (effect is independent on HO-1); - ↓ proliferation (effect is independent on HO-1, ROS, CB_1_, CB_2_, GPR55, TRPV1); - ↑ ROS	[107]
Rat	Isolated aorta (pre-constricted with U46619 ^4^ and metoxamine ^6^)	10 μmol/L (time-dependent effect)	- Vasodilation (effect is dependent on PPAR-γ and SOD, effect is independent on endothelium, NO, CB_1_, CB_2_, TRPV1)	[106]
Rat	Isolated small mesenteric arteries (pre-constricted with phenylephrine ^6^)	0.1–30 μmol/L	- Vasodilation (effect is independent on CB_1_, CB_2_, endothelium, TRPV1)	[48]
Rat	Isolated perfused heart	30 μmol/L	- ↓ HR, contractility; - arrythmias and asystole	[108]
Rat	Isolated perfused heart	9–100 μmol/L	- ↑ (slight) HR, pulse pressure, coronary blood flow	[110]
Rat	Isolated left atrium	0.001–30 μmol/L	- ↓ contractility	[52]
Rat	Isolated ventricular cardiomyocytes	0.01–10 μmol/L	- ↓ contractility	[109]

^1^ concerning only cardiovascular system; ^2^ effects observed with at least one of the tested concentrations; ^3^ patients with cancer or inflammatory bowel disease; ^4^ thromboxane receptor agonist; ^5^ patients with lung carcinoma; ^6^ α_1_-adrenergic receptor agonist; ↑/↓/↔—increase/decrease/no change; abbreviations: abbreviations: Abn-CBD: Abnormal-cannabidiol; AEA: Anandamide; Akt: Protein kinase B; CB_1, 2_: Cannabinoid receptor type 1, 2; COX: Cyclooxygenase; CREB: cAMP response element-binding protein; EP_4_: Prostaglandin E receptor 4; ERK1/2: Extracellular signal-regulated kinase 1/2; GPR55: G-protein coupled receptor 55; HO-1: Heme oxygenase-1; HR: Heart rate; IP: Prostacyclin receptor; JNK: c-Jun N-terminal kinase; NF-кB: Nuclear factor κB; NOS: Nitric oxide and its synthase; PGE: Prostaglandin E; PPAR-γ: Peroxisome proliferator-activated receptor γ; p70S6K: Ribosomal protein S6 kinase; ROS: Reactive oxygen species; SOD: Superoxide dismutase; STAT5: Signal transducer and activator of transcription 5; TRP: Transient receptor potential; TRPV1: Transient receptor potential vanilloid subfamily member 1

To conclude, under physiological conditions, CBD has minimal impact on the cardiovascular system. Therefore, it does not carry an increased cardiovascular risk, such as THC [36]. In addition, CBD can attenuate some THC-induced effects in cardiovascular system. In rabbits, THC (3 mg/kg) and CBD (25 mg/kg) given alone (i.v.) decreased HR by about 40–50% and 10–20%, respectively. However, pretreatment with CBD (25 mg/kg) reduced magnitude and duration of THC-induced bradycardia [103]. In humans, vaporized THC (8 mg) led to an intoxication and tachycardia. Low doses of CBD (4 mg) when combined with THC enhanced its intoxicating effects (but did not influence increase in HR), while high doses of CBD (400 mg) attenuated both THC-induced intoxication and tachycardia [7]. Thus, proportions of THC and CBD might be essential for protective influence of CBD on cardiovascular effects caused by THC. In another study, oral CBD (200, 400 and 800 mg) does not alter the subjective, reinforcing and cardiovascular effects of smoked cannabis (1/2 of cigarette containing ~800 mg of cannabis; 5.3–5.8% THC) [93]. CBD (1 mg/kg) and THC (0.5 mg/kg) mixture (p.o.) did not prevent tachycardia (in contrast to anxiety and other marijuana-like effects) induced by THC given alone (0.5 mg/kg; p.o.) in humans [77]. In a clinical study with THC (5 and 15 mg), and low (5.4 mg THC, 5.0 mg CBD) and high (16.2 mg THC, 15.0 mg CBD) doses of Sativex^®^ no significant CBD-induced modulation of tachycardia and other physiological effects evoked by THC was observed [111]. On the other hand, CBD at equimolar consternations diminished THC-induced increase of HR, and decrease of pulse pressure and coronary blood flow in isolated rat hearts [110]. 

## 4. Effects of Cannabidiol on the Cardiovascular System under Pathological Conditions

The endocannabinoid system does not seem to be significant for cardiovascular regulation under physiological conditions, as both FAAH inhibitors and CB_1_ receptor antagonists do not significantly affect blood pressure in normotensive animals [19,20]. This situation changes in pathological conditions when activation of the endocannabinoid system is often observed [20,27]. Such activation may be protective or detrimental, e.g., endocannabinoid-induced vasorelaxation is beneficial in arterial hypertension, but deleterious in septic shock or portal hypertension [20,27]. Pathological states might also modify action of administered cannabinoids, e.g., depressor response to THC is stronger in hypertensive than in normotensive patients [20]. Moreover, the influence of cannabinoids on the cardiovascular system may be achieved through their modulating effect on immune processes or redox balance occurring via cannabinoid and non-cannabinoid receptors. Cannabinoids can cause oxidative stress and proinflammatory effects (mainly through CB_1_ receptors) as well as antioxidative and anti-inflammatory effects (mainly through CB_2_ receptors) [27.31]. Cannabidiol is known to possess antioxidant (with some exceptions, see above) and anti-inflammatory properties [6,30,31,32,33]. Thus, it might have therapeutic potential in the treatment of different cardiovascular diseases, as the oxidative stress and inflammation are essential parts of their pathogenesis.

### 4.1. Stress-Induced Cardiovascular Changes

Cannabidiol may act as an anti-anxiety agent under stress conditions both in animal models [55,82,87] and in humans [80]. Stressful situations are associated with increases in blood pressure and heart rate, whereas the meta-analysis by Sultan et al. [24] showed that CBD eliminates both. Decreases in elevated MBP and HR in rat stress models were observed after administration of CBD intraperitoneally [55,82] as well as centrally into the cisterna magna [87] or BNST [90] (Table 4). Interestingly, the injection of CBD into BNST only produced these effects in the contextual conditioned fear in rats [90]. In acute restraint stress, CBD administered to BNST (at the same doses) did not affect MBP and even enhanced restraint-induced increase of HR [92]. It has been demonstrated that CBD affects stress-related changes in the cardiovascular system via 5-HT_1A_ receptors [55,90,92]. In humans undergoing various types of stress, conflicting results have been reported. Thus, CBD (300 or 600 mg, p.o.) did not affect blood pressure and/or HR increased by simulated public speaking [80,112]. Conversely, acute administration of CBD (600 mg, p.o.) decreased or tended to decrease blood pressure (and other hemodynamic parameters, see Table 4) and increased HR during different stressful conditions such as mental arithmetic test, isometric hand-grip test or cold stress [99]. In another study, the same dose of CBD after both acute and chronic treatment (for seven days) slightly reduced SBP, but did not influence HR (and other cardiovascular parameters, see Table 4) during isometric handgrip stress exercise. Thus, the tolerance (observed under resting conditions, see above) for hypotensive effect of CBD during stress does not develop. In addition, it has been demonstrated that repeated CBD dosing decreased arterial stiffness and improved endothelial function [97]. To sum up, CBD, aside from its potential anxiolytic action, may exhibit additionally beneficial hemodynamic effects in stressful situations. However, these protective effects may be, at least in part, the result of CBD anti-anxiolytic properties. It is worth noting that stress is probably the state in which the influence of CBD on the hemodynamic parameters is most pronounced.

### 4.2. Arterial Hypertension 

Arterial hypertension is associated with changes in the endocannabinoid system (e.g., increase of AEA in plasma) which may indicate its activation. Cannabinoids administered to hypertensive animals often have changed hemodynamic responses—the hypotensive phase appears or is intensified. Inhibition of FAAH can exert a hypotensive effect, which depends on the age of the animals and experimental model of hypertension. Endocannabinoids has also shown a modulating effect of on oxidative stress and inflammation in hypertension, which are the important part of the pathogenesis of this disease [19,20,27,113,114]. Taking into account both the vasorelaxant effect, modulation of inflammatory and oxidative processes and endocannabinoid metabolism [23,27,30,31,71], certain benefits may be expected from their use in hypertension. 

Thus far, studies have been conducted on two rat models of hypertension (Table 4)—in spontaneously hypertensive rats (SHR; model of primary hypertension) and deoxycorticosterone acetate-salt-induced hypertension (DOCA-salt; model of secondary hypertension) [48,52,96]. Studies on isolated small mesenteric arteries have shown opposite effects of CBD in these two models. In the former, the vasorelaxant action of CBD was reduced, while in the latter enhanced [48]. These opposite effects may result from different pathogenesis and changes in the endocannabinoid system in employed hypertension models [20]. In addition, there were also some differences in mechanism of vasodilatory action of CBD in these two models (see Table 4) [48]. Similarly to the SHR, the vasodilatory effect of CBD in isolated pulmonary arteries was reduced in patients with hypertension [48]. However, no change in vasorelaxant response to CBD was observed in isolated mesenteric arteries of hypertensive subjects [56].

In conscious rats with spontaneous hypertension, i.p. administration of CBD increased blood pressure in the first minutes after injection slightly stronger than in normotensive control [52]. Thus, it may results from impaired vasodilatory action of CBD revealed in SHR [48]. However, hemodynamic effects in pithed and vagotomised SHR (increase in SBP and HR and decrease in DBP) were comparable to controls [52]. In urethane anaesthetised SHR, rapid i.v. administration of CBD induced a stronger Bezold-Jarisch reflex than in control animals [52]. During two-week CBD administration to SHR and DOCA-salt rats no significant effect on blood pressure and heart rate was observed. At the same time, however, a decrease in oxidative stress markers in the plasma and heart of these animals was found [96]. In conclusion, the studies conducted so far do not reveal hypotensive action of CBD in hypertension, although this compound exhibit antioxidative properties in this disease. 

### 4.3. Myocardial Ischemia/Infarction, Cardiomyopathies, Myocarditis

The high cardioprotective potential of CBD has been postulated previously (Table 4). Several studies indicate beneficial effects in myocardial ischemia/infarction, which was obtained in experimental animals by ligating the left anterior descending artery in rats [49,105,115] or the left circumflex coronary artery in rabbits [116]. Administration of CBD before ligation of the coronary artery and immediately before reperfusion reduced the infarct size. Additionally, CBD administered before the induction of ischemia reduced the number of ventricular arrhythmias and decreased collagen-induced platelet aggregation. The antiarrhythmic effect of CBD might be mediated through the inhibition of release of arrhythmogenic substances from platelets [105]. Another study revealed that CBD antiarrhythmic effect against ischemia/reperfusion-induced ventricular arrhythmias is dependent on adenosine A_1_ receptors [49]. In rabbits, CBD administered before the experimentally induced acute myocardial infarction reduced infarct size, increased blood flow in the perfusion-defective region, diminished cardiac troponin I levels in blood and decreased myocellular apoptosis. In addition, CBD improved the left ventricular function and protected against reperfusion injury, which was associated with reduced leukocyte infiltration in the heart [116]. Chronic treatment with CBD also diminished infarct size and cardiac leukocyte infiltration in rat model of ischemia and reperfusion. These effects were associated with decreased serum interleukin 6 levels. However, reduction of infarct size by CBD were observed only in an in vivo, but not in the isolated hearts. Thus, cardioprotective effects of CBD in myocardial infarction seem not to be direct and might result from its anti-inflammatory properties [115]. 

Cannabidiol also exhibits potential beneficial effects in another cardiac disorders (Table 4). For example, it attenuates doxorubicin (anthracycline anticancer antibiotic) cardiotoxicity in rats. Chronic administration of CBD over four weeks lessened doxorubicin-induced histopathological changes in heart and elevations of serum markers of myocardial damage—creatine kinase and troponin T. Cardioprotective action of CBD was associated with diminished cardiac malondialdehyde, nitric oxide, tumour necrosis factor-α (TNF-α) and calcium ion levels, increased cardiac reduced glutathione, selenium and zinc ions levels, decreased expression of NF-кB, inducible NOS and caspase-3, and enhanced survivin expression [117]. In another study, CBD treatment for five days improved doxorubicin-induced cardiac dysfunction, decreased creatine kinase and lactate dehydrogenase activity in serum (markers of cardiac injury). Similar to the previous study, treatment with CBD markedly diminished oxidative and nitrative stress, and cell death in doxorubicin-induced cardiomyopathy. In addition, CBD enhanced impaired cardiac mitochondrial function and biogenesis in this pathology [118]. In the model of experimental autoimmune myocarditis in mice, injections of CBD improved systolic and diastolic properties of the left ventricle while reducing its fibrotic remodelling, inflammation, necrosis and mononuclear infiltration. Biochemical tests confirmed alleviation of inflammation associated with decreased expression of the proinflammatory cytokines (interleukin 6 and 1β and interferon-γ) and levels of cardiac 4-hydroxynonenal and 3-nitrotyrosine (oxidative and nitrative stress markers, respectively) [119]. Cardioprotective properties of CBD have also been revealed in animal model of diabetes (see below). 

### 4.4. Stroke, Neonatal Hypoxic-Ischemic Encephalopathy, Sepsis-Associated Encephalitis

The neuroprotective properties of CBD have been shown in a wide range of animal models of neurological disorders including epilepsy, Alzheimer’s disease, Huntington’s disease, Parkinson’s disease and multiple sclerosis [11,17,30,66]. There is also some evidence (Table 4) for beneficial effects of CBD in brain disorders associated with hypoxia and/or ischemia, such as stroke and neonatal hypoxic-ischemic encephalopathy (HIE). 

Cannabidiol effects in ischemic stroke have been studied mainly in mice and rats with middle cerebral artery occlusion (Table 4). In this model of stroke, CBD administered both pre- and/or post-ischemia reduced infarct volume [50,53,120,121,122] (but not in newborn rats [123]) and improved impaired neurological and/or neurobehavioral functions [50,120,121,122,123,124]. CBD increased cerebral blood flow during the occlusion [50,53], which is consistent with the meta-analysis by Sultan et al. [24] that indicated increased CBF in mouse models of stroke after CBD administration. In addition, one study showed that CBD also suppressed a decrease in CBF due to the failure of cerebral microcirculation for 1 h after reperfusion [50]. The influence of CBD on infarct volume and CBF was mediated, at least in part, through serotonin 5-HT_1A_ receptors and was not dependent on cannabinoid receptors and vanilloid TRPV1 receptors [50,53,121]. Importantly, repeated treatment with CBD for 14 days before the occlusion did not revealed the development of tolerance to its neuroprotective properties [50]. It has been demonstrated that CBD evokes neuroprotective properties when administered even after a longer period of time after cerebral ischemia. Thus, repeated treatment with CBD from the first or third day at the latest after stroke induction improved the functional deficits, survival rates and ischemic damage [124]. Protective effects of CBD against hippocampal neurodegeneration and cognitive impairment or motor hyperactivity have been demonstrated in gerbils and rats submitted to cerebral ischemia induced by bilateral carotid artery occlusion [125,126]. Neuroprotective action of CBD in animal models of stroke were associated with decreased excitotoxicity [123], glial activation [121,123,124,126], neuronal metabolism impairment [123] and apoptosis [121,123,124]. CBD also diminished stroke-induced neuroinflammation as it reduced the number of myeloperoxidase-positive cells (neutrophils) [50,121] and decreased the expression of inflammatory factors such as TNF-α receptor 1 and NF-кB in brain [122]. In addition, CBD might alleviate post-ischemic injury via inhibition of high-mobility group box1 (HMGB1) protein. This non-histone DNA-binding protein is massively released into the extracellular space from inflammatory and necrotic cells after ischemia and induces the expression of genes associated with neuroinflammation and microglial activation. Indeed, treatment with CBD decreased plasma level of HMGB1 and the number of HMGB1-positive cells in the brain of mice submitted to focal cerebral ischemia [121,124]. It should be noted that in cerebral ischemia, CBD evoked some beneficial effects in a dose-dependent bell-shaped curve. Thus, prevention of electroencephalographic flattening was greatest with 5 mg/kg of CBD (1.25–20 mg/kg) [125] and reduction in infarct volume with 1 mg/kg of CBD (0.1–10 mg/kg) [53].

The most common cause of brain damage in neonates is perinatal hypoxic-ischemic encephalopathy caused by asphyxia. Cannabidiol is considered as promising neuroprotectant after neonatal hypoxia-ischemia and as mentioned above, in European Union, this compound has even orphan drug status in perinatal asphyxia. The largest number of evidence of protective CBD action in HIE is based on studies in newborn piglets submitted to hypoxia-ischemia [54,127,128,129,130,131] (Table 4). Beneficial effects of CBD on experimental neonatal HIE include alleviation of decreased brain activity [54,127,128,129,130,131], neuronal metabolism impairment [127,128,129,131], excitotoxicity [54,129], histopathological changes in brain [127,128], neuronal necrosis and/or apoptosis [54,129,130,131], astroglial and/or microglial activation [131], neuroinflammation [54,128,129,130,131] and oxidative stress in brain [54,130,131]. CBD can also diminish distant inflammatory lung damage associated with brain hypoxia-ischemia-induced injury [130]. In addition, CBD might diminish hypoxia-ischemia-induced decrease in blood pressure [54,127,129,130,131]. However, high dose of CBD (50 and 25 mg/kg; the most common dose in previous studies was 1 mg/kg) induced significant hypotension in some piglets (CBD 50 mg/kg in two out of four piglets and CBD 25 mg/kg in one out of four piglets). In addition, one piglet suffered fatal cardiac arrest after CBD infusion at dose of 50 mg/kg [132]. Thus, caution should be taken during treatment with high doses of CBD due to possible occurrence of cardiovascular side effects. Beneficial effects of CBD have been also revealed in mouse [133] and rat models [134,135] of neonatal HIE (Table 4). It is noteworthy, that CBD shows a broader therapeutic time window than reported for hypothermia and other neuroprotective treatments [133]. The protective action of CBD in hypoxia-ischemia-induced brain injury occurs, at least in part, through CB_2_ and 5-HT_1A_ receptors [54,130]. Studies in forebrain slices from newborn mice underwent oxygen and glucose deprivation revealed that, in addition to CB_2_ receptors, adenosine receptors (mainly A_2_) can also mediate neuroprotective effects of CBD [136]. However, some authors reported that CBD (*at low* or high doses*)* did not exhibit any neuroprotective properties in experimental models of neonatal HIE [137]. Nevertheless, CBD can enhance protective effects of hypothermia, a gold standard for treating infants with HIE [129,131]. 

The anti-inflammatory and vascular-stabilizing effects of CBD have been also revealed in the murine encephalitis induced by lipopolysaccharide (LPS) administration (Table 4). LPS evoked arteriolar and venular vasodilation, enhanced leukocyte margination, increased expression of proinflammatory TNF-α and COX-2, higher levels of oxidative stress markers (malondialdehyde and 4-hydroxynonenal) and disruption of the blood–brain barrier. Treatment with CBD alleviated almost all these LPS-induced changes (with exception of oxidative stress markers), and in addition reduced inducible NOS expression. Thus, CBD may offer an option for treating sepsis-related encephalitis and encephalopathy [138].

### 4.5. Renal and Hepatic Ischemia/Reperfusion Injury

Cannabidiol has been shown to be protective against ischemia/reperfusion injury of the kidneys and liver (Table 4). This type of damage can occur during shock and surgery or transplantation of these organs. In a rat model of renal ischemia/reperfusion injury, CBD significantly reduced histopathological changes in kidneys and decreased serum creatinine (marker of renal function). The nephroprotective effect of CBD was associated with ameliorated ischemia/reperfusion-induced oxidative and nitrative stress, inflammation and apoptosis [139]. Similar protective effects were obtained in rodents submitted to ischemia/reperfusion of the liver. Thus, CBD reduced serum transaminases (markers of liver damage) and histopathological changes, cell death, oxidative and nitrative stress, and inflammation in the liver [140,141]. It has been shown that the mechanism of this hepatoprotective action may include attenuated activation of NF-κB, p38 MAPK and JNK by CBD [141]. In vitro studies in human liver sinusoidal cells showed that CBD can attenuate TNF-α-induced expression of adhesion molecules (ICAM-1 and VCAM-1) and polymorphonuclear cells adhesion to liver sinusoidal cells. This corresponds with CBD-induced decrease of ICAM-1 expression and neutrophil infiltration in the mice liver submitted to ischemia/reperfusion injury [141]. Hepatoprotective effects of CBD seem not to be dependent on cannabinoid receptors, as they were not attenuated by CB_1_ and CB_2_ antagonists in vitro and were still presented in CB_2_ knockout mice [141]. In summary, CBD has a great therapeutic potential in preventing and alleviating ischemia/reperfusion injury of different organs such as heart, brain, kidneys and liver.

### 4.6. Cardiovascular Complications of Diabetes 

Diabetes mellitus causes many heart and blood vessel complications such as atherosclerosis, retinopathy or cardiomyopathy, which are associated with vascular endothelial dysfunction, increased inflammation and oxidative stress. CBD does not influence blood glucose in diabetic animals [142,143,144] and humans [145]. In addition, in patients with type 2 diabetes CBD did not affect glycaemic control, insulin sensitivity, lipid profile, body mass and hemodynamic parameters [145]. However, due to its antioxidative, anti-inflammatory, and vasculo-, cardio- and neuroprotective properties CBD can mitigate cardiovascular complications of diabetes (Table 4).

Disruption of the endothelial function and integrity is essential for the development of various diabetic complications. In human coronary artery endothelial cells exposed to high glucose increased mitochondrial superoxide generation, 3-nitrotyrosine formation, NF-κB activation, inducible NOS and adhesion molecules expression, transendothelial migration of monocytes and their adhesion to endothelium were indicated. CBD pretreatment attenuated all these negative effects. In addition, it also improved high glucose-induced disruption of endothelial barrier function. The protective effects of CBD in endothelial cells were CB_1_ and CB_2_ independent [146].

The influence of CBD on the function of blood vessels was studied in Zucker diabetic fatty rats (ZDF)*—*type 2 diabetes model. In vitro incubation with CBD enhanced the vasodilatory effect of acetylcholine in the isolated aorta and femoral artery, and this effect was stronger compared to normoglycaemic control animals [57,147]. Studies examining the mechanism of action in the femoral arteries have shown that CBD activates cyclooxygenase and subsequently induces production of compounds activating the vasodilatory EP_4_ prostanoid receptors. Moreover, CBD’s effect was dependent on superoxide dismutase and CB_2_ receptors. Interestingly, in ZDF femoral arteries, CBD uncovered the vasodilatory action of the CB_2_ receptor agonist HU308 (this compound without the presence of CBD showed no vasodilatory effect) [57]. Cannabidiol can also improve vasorelaxation in diabetic rats after in vivo treatment. Thus, repeated administration of CBD for seven days significantly increased vasodilation to acetylcholine in isolated mesenteric arteries (but not in aorta and femoral arteries) and this effect was sensitive to COX and NOS inhibition. In addition, CBD decreased some serum metabolic and cardiovascular biomarkers (see Table 4). However, curiously, it increased circulating levels of endothelin 1 which is inconsistent with an improvement in vascular function [144]. It is noteworthy that in humans with type 2 diabetes CBD not only did not exhibit enhanced vasodilatory properties (in isolated pulmonary arteries) [48], but CBD vasorelaxant responses were blunted (in mesenteric arteries) [144].

Treatment with cannabidiol might be beneficial in diabetic retinopathy which is characterized by increased vascular permeability and neurotoxicity. In rats with streptozotocin-induced diabetes (model of type 1 diabetes) chronic administration of CBD improved blood-retinal barrier function, reduced oxidative and nitrative stress, decreased levels of TNF-α and ICAM-1 and prevented neural cell death in the retina. In addition, CBD reduced retinal levels of vascular endothelial growth factor (VEGF) which has been correlated with the breakdown of blood-retinal barrier [142]. Conversely, CBD increased circulating VEGF in ZDF rats, thus, effects of CBD on this mediator require further investigation [144]. Protective properties of CBD in the diabetic retina may be due to inhibition of p38 MAPK activation. This protein kinase is a downstream target of oxidative stress and proinflammatory cytokines and its activation can increase vascular permeability and cell death, the key elements of diabetic retinopathy pathogenesis [142].

Another complication of diabetes is cardiomyopathy, characterized by diastolic and subsequent systolic left ventricular dysfunction. The pathogenesis of diabetic cardiomyopathy is complex and includes oxidative/nitrative stress, inflammation, cardiac fibrosis and cardiomyocyte death. In mice with streptozotocin-induced diabetes, chronically administrated CBD mitigate all these changes by inhibition of pro-inflammatory and cell death pathways (NF-κB, p38 and p38α MAPK, JNK) and enhancing prosurvival signalling pathway (Akt) [143]. Similarly, in human cardiomyocytes, CBD eliminated the adverse effects of hyperglycaemia by reducing oxidative and nitrative stress, NF-κB activation and cell apoptosis [143].

**Table 4:** Effects of cannabidiol (CBD) in cardiovascular disorders.

Species	Experimental Model/Conditions	Dosage or Concentration	Effects ^1^	References
**1. Stress-induced cardiovascular changes**				
Human	Simulated public speaking	300 mg; p.o.	- ↔ stress-induced increase in SBP	[80]
Human	Simulated public speaking in patients with social anxiety disorder	600 mg; p.o.	- ↔ stress-induced increase in SBP, DBP, HR	[112]
Human	Mental stress (mental arithmetic test), exercise stress (isometric hand-grip test) or cold stress (cold pressor test)	600 mg; p.o.	- ↓ SBP, DBP, MBP, SV, EJT, TPR, SBF - ↑ HR - ↔ CO (just before and/or during and/or after the stress test)	[99]
Human	Exercise stress (isometric hand-grip test)	600 mg; p.o.	- ↓ SBP (during the stress test), ↔ SBP (just before and after the stress test) - ↔ DBP, MBP, HR, CO, SV, EJT, TPR (just before, during and after the stress test)	[97]
		600 mg; for 7 days; p.o.	- ↓ SBP (during the stress test), ↔ SBP (just before and after the stress test) - ↔ DBP, MBP, HR, CO, SV, EJT, TPR (just before, during and after the stress test) - ↓ arterial stiffness (↓ PWV) - ↑ endothelial function (↑ FMD)	
Rat	Contextual conditioned fear	10 mg/kg; i.p.	- ↓ stress-induced increase in MBP, HR	[82]
Rat	Acute restraint stress	1; 10; 20 mg/kg; i.p.	- ↓ stress-induced increase in MBP, HR (effect is dependent on 5-HT_1A_)	[55]
Rat	Acute restraint stress	15; 30; 60 nmol; i.c.	- ↓ stress-induced increase in MBP, HR	[87]
Rat	Contextual conditioned fear	15; 30; 60 nmol; into BNST	- ↓ stress-induced increase in MBP, HR (effect is dependent on 5-HT_1A_)	[90]
Rat	Acute restraint stress	15; 30; 60 nmol; into BNST	- ↔ stress-induced increase in MBP - ↑ stress-induced increase in HR (effect is dependent on 5-HT_1A_)	[92]
**2. Arterial hypertension**				
Human	Hypertensive patients ^2^; isolated mesenteric arteries (pre-constricted with U46619 ^3^ and endothelin-1)	0.1–100 μmol/L	- ↔ vasorelaxant response	[56]
Human	Hypertensive patients ^4^; isolated pulmonary arteries (pre-constricted with U46619 ^3^)	0.1–30 μmol/L	- ↓ vasorelaxant response	[48]
Rat	SHR (model of primary hypertension); conscious	10 mg/kg; i.p.	- ↑ SBP, DBP (slightly stronger than in normotensive control) - ↔ HR	[52]
	SHR (model of primary hypertension); urethane anaesthetised, pithed and vagotmised	1; 3; 30 mg/kg; i.v.	- ↑ SBP, HR - ↓ DBP (comparable with normotensive control)	
	SHR (model of primary hypertension); urethane anaesthetised	3; 10; 30 mg/kg; i.v. (rapid)	- ↓ SBP, DBP, HR (Bezold-Jarisch reflex induced via TRPV1 receptors; stronger than in normotensive control) - ↓ Bezold-Jarisch reflex induced by 5-HT_3_ (but not TRPV1) activation (comparable with normotensive control)	
	SHR (model of primary hypertension); isolated left atrium	0.001–30 μmol/L	- ↓ contractility (slightly less than in normotensive control)	
Rat	SHR (model of primary hypertension)	10 mg/kg; for 14 days; i.p.	- ↔ SBP, DBP, HR - ↓ oxidative stress markers in plasma (↓ carbonyl groups) and in heart (↓ 4-HHE)	[96]
	DOCA-salt (model of secondary hypertension)		- ↔ SBP, HR - ↓ oxidative stress markers in plasma and heart (↓ MDA)	
Rat	SHR (model of primary hypertension); isolated small mesenteric arteries (pre-constricted with phenylephrine)	0.1–30 μmol/L	- ↓ vasorelaxant response (vasodilation is dependent on endothelial CB_1_; independent on endothelium, CB_2_, TRPV1)	[48]
	DOCA-salt (model of secondary hypertension); isolated small mesenteric arteries (pre-constricted with phenylephrine)		- ↑ vasorelaxant response (vasodilation is dependent on endothelium, CB_2_ and endothelial CB_1_; independent on TRPV1)	
**3. Myocardial ischemia/infarction, cardiomyopathies, myocarditis**				
Rabbit	LCx occlusion (90 min) + reperfusion (30 h); model of myocardial ischemia/infarction	0.1 mg/kg; 10 min before occlusion and 10 min before reperfusion; i.v.	- ↓ blood troponin I - ↓ dysfunction of left ventricle (increased SV, CO, EF, systolic wall thickening) - ↑ blood flow in the in the perfusion-defective region - ↓ infarct size - ↓ myocardial oedema and microvascular obstruction - ↓ cardiac neutrophil infiltration - ↓ apoptosis in heart	[116]
Rat	LAD occlusion (30 min) + reperfusion (7 days); model of myocardial ischemia/infarction	5 mg/kg; before occlusion and every 24 h thereafter for 7 days; i.p.	- ↔ shortening fraction in echocardiography - ↓ infarct size - ↓ leukocyte infiltration in heart - ↓ serum IL-6 - ↔ serum CRP, TNF-α - ↔ HR	[115]
	LAD occlusion in isolated heart (45 min) + reperfusion (45 min); model of myocardial ischemia/infarction	5 mg/kg; 24 h and 1 h before heart isolation; i.p.	- ↔ infarct size - ↔ contractility - ↔ coronary flow	
Rat	LAD occlusion (30 min) + reperfusion (2 h); model of myocardial ischemia/infarction	10 or 50 µg/kg; 10 min before occlusion; i.v.	- ↓ MBP - ↔ HR - ↓ arrhythmias - ↓ infarct size - ↓ platelet aggregation (comparable with sham group) - ↔ mast cells degranulation in heart	[105]
		50 µg/kg; 10 min before reperfusion; i.v.	- ↓ MBP - ↔ HR - ↔ arrhythmias - ↓ infarct size - ↔ platelet aggregation - ↔ mast cells degranulation in heart	
Rat	LAD occlusion (6 min) + reperfusion (6 min); model of myocardial ischemia/infarction	50 µg/kg; 10 min before occlusion; i.v.	- ↓ arrhythmias (dependent on A_1_) - ↔ MBP, HR	[49]
Rat	Doxorubicin-induced cardiomyopathy	5 mg/kg; for 4 weeks; i.p.	- ↓ serum troponin T and CK-MB - ↓ histopathological changes in heart - ↓ oxidative and nitrative stress in heart - ↓ inflammation in heart - ↓ apoptosis in heart - ↓ NF-κB expression in heart - ↓ Ca and ↑ Zn and Se in heart	[117]
Mouse	Doxorubicin-induced cardiomyopathy	10 mg/kg; for 5 days; i.p.	- ↓ serum CK and LDH - ↓ cardiac dysfunction - ↓ oxidative and nitrative stress in heart - ↓ impaired cardiac mitochondrial function and biogenesis - ↓ activation of MMP2 and MMP9 in heart - ↓ cell death in heart - ↓ inflammation in heart	[118]
Mouse	Experimental autoimmune myocarditis	10 mg/kg; for 46 days; i.p.	- ↓ cardiac dysfunction (improved systolic function and reverted diastolic dysfunction and myocardial stiffness) - ↓ myocardial fibrosis - ↓ oxidative and nitrative stress in heart - ↓ inflammation in heart - ↓ mononuclear cell infiltration in heart - ↓ necrosis in heart	[119]
****4. Stroke, neonatal hypoxic-ischemic** encephalopathy, sepsis-related encephalitis**				
Piglet (newborn)	Hypoxia and carotid arteries occlusion (20 min) + post-HI period (6 h); model of neonatal HIE	0.1 mg/kg; 15 min and 240 min after HI; i.v.	- ↓ cerebral hemodynamic and metabolic impairment - ↑ brain activity (EEG amplitude) - ↓ seizures - ↓ loss of neurons and neuron degeneration in cortex and hippocampus - ↓ blood troponin T - ↓ HI-induced decrease in MBP and increase in HR - ↓ HI-induced blood gases and respiratory abnormalities	[127]
Piglet (newborn)	Hypoxia and carotid arteries occlusion (40 min) + post-HI period (6 h); model of neonatal HIE	1 mg/kg; 30 min after HI; i.v.	- ↓ impairment in brain activity - ↓ neuronal necrosis in cortex - ↑ number of astrocytes in cortex - ↓ excitotoxicity in cortex - ↓ oxidative stress in cortex - ↓ neuroinflammation in cortex - ↓ HI-induced decrease in MBP - ↔ blood pCO2 and decreased blood pH - ↔ CO (effects are dependent on CB_2_ and 5-HT_1A_)	[54]
Piglet (newborn)	Hypoxia and carotid arteries occlusion (20 min) + post-HI period (6 or 72 h); model of neonatal HIE	0.1 mg/kg; 15 min and 240 min after HI; i.v.	- ↑ brain activity (EEG amplitude) - ↓ impairment in brain metabolism - ↓ impairment in neurobehavioral functions - ↓ histopathological changes in brain - ↓ TNF-α-positive cells in brain - ↓ S100B (astrocytic injury marker) and neuronal specific enolase in CSF	[128]
Piglet (newborn)	Hypoxia and carotid arteries occlusion (40 min) + post-HI period (6 h); model of neonatal HIE	1 mg/kg; 30 min after HI; i.v.	- ↓ impairment in brain activity - ↓ neuronal necrosis in cortex - ↓ neuronal metabolism impairment in cortex - ↓ apoptosis in cortex - ↓ excitotoxicity in cortex - ↓ oxidative stress in cortex - ↓ neuroinflammation in cortex - ↓ HI-induced decrease in MBP - ↔ blood pCO2 and decreased blood pH - ↔ CO - ↑ beneficial effects of hypothermia on HI-induced toxicity, neuroinflammation, oxidative stress and neuron damage in cortex	[129]
Piglet (newborn)	Hypoxia + post-hypoxic period (9,5 h); model of neonatal HIE	1 mg/kg; 30 min after hypoxia; i.v.	- ↔ neuropathological changes in cortex, hippocampus, white matter, and cerebellum - ↔ oxidative stress markers in urine - ↔ neuroinflammation in cortex and hippocampus - ↔ apoptosis in cortex - ↔ excitotoxicity in hippocampus - ↔ neuronal metabolism impairment in cortex - ↔ blood haemoglobin, lactate, glucose and troponin T - ↔ urine NGAL (↓ urine NGAL when hypothermia was applied) - ↔ S100B (astrocytic injury marker) in CSF - ↔ HI-induced decrease in MBP and increase in HR - ↔ HI-induced blood gases abnormalities - ↔ beneficial effects of hypothermia	[137]
Piglet(newborn)	Hypoxia + post-hypoxic period (6 h); model of neonatal HIE	1 mg/kg; 30 min after hypoxia; i.v.	- ↓ HI-induced decrease in MBP - ↑ brain activity (EEG amplitude) - ↓ death of neurons in brain↓ neuroinflammation in brain - ↓ oxidative stress in brain - ↓ lung oedema and histological changes and inflammation in lungs(all above effects are dependent on 5-HT_1A_) - ↓ gas exchange in lungs and ↑ total lung capacity (effects are independent on 5-HT_1A_) - ↔ oxidative stress in lungs - ↔ CO and blood gases	[130]
Piglet (newborn)	Hypoxia + post-hypoxic period (9,5 h); model of neonatal HIE	50 mg/kg ^5^; 30 min after hypoxia; i.v. over 15 min.	- ↓ MBP (significant) - ↔ HR, body temperature, blood haemoglobin, lactate and troponin T - ↔ S100B (astrocytic injury marker) in CSF - ↔ neuropathological changes in brain - ↔ excitotoxicity in brain - ↔ neuronal metabolism impairment in brain	[132]
Piglet(newborn)	Hypoxia and carotid arteries occlusion (20 min) + post-HI period (54 h); model of neonatal HIE	1 mg/kg; 0.5, 24 and 48 h after HI; i.v.	- ↑ brain activity (EEG amplitude) - ↓ microglial activation in brain - ↔ excitotoxicity in brain - ↔ neuronal metabolism impairment in brain - ↔ neuroinflammation in brain - ↔ apoptosis in brain - ↔ oxidative stress in brain - ↔ astroglial activation - ↓ HI-induced decrease in MBP - ↔ HR, CO - ↔ blood gases - ↑ brain activity and ↓ microglial activation, excitotoxicity, neuronal metabolism impairment and inflammation in brain when hypothermia was applied	[131]
Gerbil	Carotid arteries occlusion (10 min) + reperfusion (7 days); model of stroke	1.25; 2.5; 5; 10 or 20 mg/kg; 5 min after occlusion; i.p.	- ↓ EEG flattening - ↑ survival of neurons in the CA_1_ region of the hippocampus - ↓ hyperlocomotion 1 day after occlusion - ↔ rectal temperature 1 h after occlusion	[125]
Rat	MCA occlusion (90 min) + reperfusion (2 days); model of stroke	5 mg/kg; at the onset of occlusion; i.v. + 20 mg/kg; 12 h after occlusion; i.p.	- ↓ infarct volume in brain - ↑ behavioral parameters - ↔ BP - ↔ blood gases and blood glucose - ↔ rectal temperature	[120]
Rat (newborn)	Hypoxia (120 min) and left carotid artery electrocoagulation + post-HI period (7 or 30 days); model of neonatal HIE	1 mg/kg; 10 min after hypoxia; s.c.	- ↑ neurobehavioral function - ↓ infarct volume - ↓ histopathological changes in brain - ↓ excitotoxicity - ↓ neuronal metabolism impairment - ↓ neuronal loss - ↓ oxidative stress - ↓ neuroinflammation	[134]
Rat (newborn)	MCA occlusion (3 h) + reperfusion (1 week or 1 month); model of neonatal stroke	5 mg/kg; 15 min after occlusion; i.p.	- ↑ neurobehavioral function - ↔ infarct volume - ↓ perilesional gliosis volume - ↓ neuronal loss and apoptosis - ↓ excitotoxicity - ↓ neuronal metabolism impairment - ↓ astrocyte dysfunction - ↓ microglial proliferation and activation	[123]
Rat	MCA occlusion (1 h) + reperfusion (1 day); model of stroke	50, 100 or 200 ng; for 5 days before occlusion; i.c.v.	- ↓ infarction volume in total of cerebral hemisphere, cortex, piriform cortex amygdala and striatum - ↓ TNF receptor 1 and NF-кB in total of cerebral hemisphere, cortex and striatum	[122]
Rat (newborn)	Hypoxia (112 min) and left carotid artery electrocoagulation + post-HI period (30 days); model of neonatal HIE	1 mg/kg; 10 min after hypoxia; s.c.	- ↓ impairment of myelination in white matter and cortex - ↓ impairment in neurobehavioral performance - ↔ BDNF and GDNF expression in cortex 7 days after HI	[135]
Mouse	MCA occlusion (4 h) + reperfusion (20 h); model of stroke	0.1; 1; 3 or 10 mg/kg; immediately before occlusion and 3 h after onset of the occlusion; i.p.	- ↓ infarct volume (effect is dependent on 5-HT_1A_; independent on CB_1_ and TRPV1) - ↑ CBF during occlusion (effect is dependent on 5-HT_1A_) - ↔ MBP, HR (2 h after onset of the occlusion) - ↔ blood gases and haematocrit before reperfusion	[53]
Mouse	MCA occlusion (4 h) + reperfusion (20 h or 3 days); model of stroke	3 mg/kg; immediately before occlusion and 3 h after onset of the occlusion; i.p.	- ↓ infarct volume (20 h or 3 days after occlusion) - ↔ blood gases, haematocrit, blood K and Na (before reperfusion) - ↔ MBP, HR (before reperfusion) - ↔ rectal temperature (before reperfusion) - ↑ CBF during occlusion and for 1 h after occlusion - ↓ MPO activity in brain (1 and 20 h after occlusion; effect is independent on CB_1_ and CB_2_) - ↓ MPO-positive cells in striatum (20 h or 3 days after occlusion) - ↑ motor coordination (3 days after occlusion)	[50]
	MCA occlusion (4 h) + reperfusion (20 h); model of stroke	0.1; 1 or 3 mg/kg; immediately before occlusion and 3 h after onset of the occlusion; i.p.	- ↓ infarct volume (effect is independent on CB_1_ and CB_2_)	
		3 mg/kg; immediately before occlusion or 3, 4, 5, 6 h after onset of the occlusion; i.p.	- ↓ infarct volume - ↔ excitotoxicity in the cortex during 2 h after onset of the occlusion (CBD given immediately before the occlusion) - ↓ MPO activity in brain (CBD given 6 h after onset of the occlusion)	
Mouse	MCA occlusion (4 h) + reperfusion (20 h); model of stroke	0.1; 1 or 3 mg/kg; immediately before occlusion and 3 h after onset of the occlusion; i.p.	- ↓ infarct volume (effect is independent on CB_1_ and CB_2_; dependent on 5-HT_1A_) - ↑ CBF during occlusion (effect is dependent on 5-HT_1A_) - ↔ blood gases, haematocrit, blood K and Na before reperfusion - ↔ CB_1_ expression in cortex, striatum and hypothalamus	[50]
		3 mg/kg; for 14 days before occlusion + immediately before occlusion and 3 h after onset of the occlusion; i.p.	- ↓ infarct volume (dependent on 5-HT_1A_) - ↑ CBF during occlusion (effect is dependent on 5-HT_1A_) (effects are comparable to those observed in the group not treated with CBD for 14 days) - ↔ rectal temperature at 1 h after onset of the occlusion - ↔ blood gases, haematocrit, blood K and Na before reperfusion - ↔ CB_1_ expression in cortex, striatum and hypothalamus	
Mouse	MCA occlusion (4 h) + reperfusion (20 h); model of stroke	0.1; 1 or 3 mg/kg; immediately before occlusion and 3 h after onset of the occlusion; i.p.	- ↔ MBP, HR, pH, pCO_2_, haematocrit, Na, K, blood glucose, body temperature (before reperfusion) - ↓ infarct volume (effect is independent on CB_1_ and CB_2_) - ↓ MPO activity in brain (effect is independent on CB_1_ and CB_2_) - ↓ plasma HMGB1 - ↑ neurological function and motor coordination	[121]
	MCA occlusion (4 h) + reperfusion (3 days); model of stroke	3 mg/kg; immediately before occlusion and 3 h after onset of the occlusion; i.p.	- ↓ plasma HMGB1 - ↓ HMGB1- and MPO-positive cells in brain - ↓ apoptosis in brain - ↓ glial activation in brain - ↑ neurological function and motor coordination	
Mouse	MCA occlusion (4 h) + reperfusion (14 days); model of stroke	3 mg/kg; for 14, 12 or 10 days from day 1, 3 or 5, respectively; i.p.	- ↓ impaired neurological functions - ↓ impaired motor coordination - ↑ survival rate - ↓ apoptosis in brain - ↓ number of microglial cells (but not astrocytes) expressing HMGB1 - ↓ plasma HMGB1 (effects for CBD administered from day 1 and 3, but not from day 5)	[124]
Mouse(newborn)	Forebrain slices underwent oxygen and glucose deprivation;in vitro model of neonatal HIE	100 μmol/L	- ↓ necrotic and apoptotic cell death - ↓ excitotoxicity - ↓ inflammation (effects are dependent on CB_2_ and A_2_; independent on CB_1_; excitotoxicity is also A_1_ dependent)	[136]
Mouse	Lipopolysaccharide-induced encephalitis;model of sepsis-related encephalitis	3 mg/kg; i.v.	- ↓ arteriolar and venular vasodilation - ↓ leukocyte margination - ↓ blood brain barrier integrity - ↓ inflammation in brain↔ oxidative stress in brain - ↔ BP - ↔ blood gases	[138]
Mouse	Carotid arteries occlusion (17 min) + reperfusion (7 days); model of stroke	3, 10 or 30 mg/kg; 30 min before and 3, 24 and 48 h after occlusion; i.p.	- ↓ hippocampal neurodegeneration - ↑ spatial learning performance - ↓ astroglial response	[126]
Mouse(newborn)	Hypoxia (90 min) and left carotid artery electrocoagulation + post-HI period (7 days); model of neonatal HIE	1 mg/kg; 15 min, 1, 3, 6, 12, 18 or 24 h after HI; s.c.	- ↓ volume loss of ipsilateral hemisphere - ↓ histopathological changes - ↓ apoptosis - ↓ astrogliosis - ↓ microglial activation (effects for CBD administered up to 18 h after HI) - ↓ histopathological changes - ↓ microglial activation (effects for CBD administered 24 h after HI)	[133]
**5. Renal and hepatic ischemia/reperfusion injury**				
Human	Human liver sinusoidal endothelial cells (HLSEC) stimulated with TNF-α	1 μmol/L	- ↓ adhesion molecules ICAM-1 and VCAM-1 - ↓ polymorphonuclear cells adhesion to HLSEC (effects are independent on CB_1_ and CB_2_)	[141]
Rat	Pedicle of the left hepatic lobe occlusion (30 min) + reperfusion (72 h)	5 mg/kg; 1 h after occlusion and every 24 h thereafter for 2 days; i.v.	- ↓ serum alanine transaminase - ↓ histopathological changes in liver - ↓ oxidative and nitrative stress in liver - ↓ inflammation in liver - ↓ apoptosis in liver - ↓ expression of NF-κB in liver	[140]
Rat	Renal vascular pedicles occlusion (30 min) + reperfusion (24 h)	5 mg/kg; 1 h before and 12 h after occlusion; i.v.	- ↓ serum creatinine - ↓ histopathological changes in kidney - ↓ oxidative and nitrative stress in kidney - ↓ inflammation in kidney - ↓ apoptosis in kidney - ↓ expression of NF-κB in kidney	[139]
Mouse	Hepatic artery and portal vein occlusion (1 h) + reperfusion (2, 6 or 24 h)	3 or 10 mg/kg; 2 h before or 90 min after occlusion; i.p.	- ↓ serum alanine and aspartate transaminase (effect is independent on CB_2_) - ↓ histopathological changes in liver - ↓ cell death in liver - ↓ inflammation in liver - ↓ ICAM-1 in liver - ↓ neutrophil infiltration in liver - ↓ NF-κB activation in liver - ↓ p38 MAPK and JNK activation in liver - ↓ oxidative and nitrative stress in liver - ↓ mitochondrial dysfunction in liver	[141]
**6. Diabetes and its cardiovascular complications**				
Human	Human coronary artery endothelial cells (HCAEC) exposed to high glucose	1.5–6 μmol/L; 48 h	- ↓ adhesion molecules ICAM-1 and VCAM-1 - ↓ monocyte-endothelial adhesion - ↓ transendothelial migration of monocytes - ↓ disruption of endothelial barrier function - ↓ NF-κB activation (above effects are independent on CB_1_ and CB_2_) - ↓ oxidative and nitrative stress	[146]
Human	Human cardiomyocytes exposed to high glucose	4 μmol/L; 48 h	- ↓ oxidative and nitrative stress - ↓ apoptosis (mediated via modulation of Akt activity) - ↓ NF-κB activation	[143]
Human	Type 2 diabetic patients ^2^; isolated mesenteric arteries (pre-constricted with U46619 ^3^ and endothelin-1)	0.1–100 μmol/L	- ↓ vasorelaxant response	[56]
Human	Type 2 diabetic patients	100 mg; twice a day; for 13 weeks; p.o.	- ↔ SBP, DBP, HR - ↔ blood glucose, glycaemic control and insulin sensitivity - ↔ lipid profile (HDL-cholesterol, LDL-cholesterol, total-cholesterol, triglycerides, apolipoprotein A and B) - ↔ body mass - ↔ adiponectin, ↓ resistin, ↑ GIP in blood	[145]
Human	Type 2 diabetic patients ^4^; isolated pulmonary arteries (pre-constricted with U46619 ^3^)	0.1–30 μmol/L	- ↓ vasorelaxant response	[48]
Rat	Streptozotocin-induced diabetes (model of type 1 diabetes)	10 mg/kg (every 2 days); for 1, 2 or 4 weeks; i.p.	- ↔ blood glucose - ↔ body weight - ↓ blood-retinal-barrier breakdown - ↓ neural cell death in the retina - ↓ oxidative and nitrative stress in the retina - ↓ inflammation in the retina - ↓ VEGF in the retina - ↓ activation of p38 MAPK in the retina	[142]
Rat	ZDF (model of type 2 diabetes); isolated aorta and femoral artery	10 µmol/L; 2 h	- ↑ vasorelaxant response to acetylcholine (stronger than in normoglycaemic control)	[147]
Rat	ZDF (model of type 2 diabetes); isolated femoral artery	10 μmol/L; 2 h	- ↑ vasorelaxant response to acetylcholine (stronger than in normoglycaemic control; effect is dependent on SOD, COX, EP_4_ and CB_2_; independent on endothelium, NO, H_2_O_2_, CB_1_, Abn-CBD receptors and PPAR-γ) - uncovering of the vasorelaxant response to CB_2_ agonist	[57]
Rat	ZDF (model of type 2 diabetes)	10 mg/kg; for 7 days; i.p.	- ↑ vasorelaxant response to acetylcholine (but not to sodium nitroprusside) in isolated mesenteric arteries (effect is dependent on COX and NO) - ↔ vasorelaxant response to acetylcholine and sodium nitroprusside in aorta and femoral artery - ↔ blood glucose - ↓ body weight gain - ↓ C peptide, insulin, leptin, ICAM-1 in serum - ↔ GLP-1, glucagon, MCP-1, pancreatic polypeptide, amylin, GIP, IL-6, TNF-α, peptide YY, vWF, PAI-1 in serum - ↑ VEGF and endothelin-1 in serum	[144]
Mouse	Streptozotocin-induced diabetes (model of type 1 diabetes)	1, 10 or 20 mg/kg; for 4 or 11 weeks; i.p.	- ↔ blood glucose, pancreas insulin content - ↔ body weight - ↓ left ventricular dysfunction - ↓ oxidative and nitrative stress in heart - ↓ inflammation and NF-κB activation in heart - ↓ apoptosis and MAPK activation in heart - ↓ myocardial fibrosis - ↓ activation of NF-κB, JNK, p38 and p38α MAPK in heart - ↑ activation of Akt in heart	[143]

^1^ Effects observed with at least one of the tested doses/concentrations; ^2^ patients with cancer or inflammatory bowel disease; ^3^ thromboxane receptor agonist; ^4^ patients with lung carcinoma; ^5^ if significant side effects occurred, the dose was reduced stepwise to 25 and 10 mg/kg. ↑/↓/↔—increase/decrease/no change; abbreviations: 4-HHE: 4-Hydroxyhexenal; 5-HT_1A, 3_: Serotonin receptors type 1A, 3; Abn-CBD: Abnormal-cannabidiol; A_1, 2_: Adenosine receptor type 1, 2; Akt: Protein kinase B; BDNF: Brain-derived neurotrophic factor; BNST: Bed nucleus of the stria terminalis; BP: Blood pressure; CB_1, 2_: Cannabinoid receptor type 1, 2; CBF: Cerebral blood flow; CK, CK-MB: Creatine kinase and its cardiac isoenzyme; CO: Cardiac output; COX: Cyclooxygenase; CRP: C-reactive protein; CSF: Cerebrospinal fluid; DBP: Diastolic blood pressure; DOCA-salt: Deoxycorticosterone acetate-salt; EEG: Electroencephalographic; EF: Ejection fraction; EJT: Left ventricular ejection time; FMD: Flow mediation dilatation; GDNF: Glial-derived neurotrophic factor; GIP: Glucose-dependent insulinotropic peptide; GIP: Glucose-dependent insulinotropic peptide; GLP-1: Glucagon-like peptide-1; HDL: High-density lipoprotein; HI: Hypoxia-ischemia; HIE: Hypoxic-ischemic encephalopathy; HMGB1: High mobility group box 1; HR: Heart rate; i.c.: Intracisternally; i.c.v.: Intracerebroventricular; i.p.: Intraperitoneally; i.v.: Intravenously; ICAM-1: Intercellular adhesion molecule 1; IL-6: interleukin 6; JNK: c-Jun N-terminal kinase; LAD: Left anterior descending artery; LCx: Left circumflex coronary artery; LDH: Lactate dehydrogenase; LDL: Low-density lipoprotein; MAPK: Mitogen-activated protein kinases; MBP: mean blood pressure; MCA: Middle cerebral artery; MCP-1: Monocyte chemoattractant protein-1; MDA: Malondialdehyde; p.o.: Per os, orally; MMP2, 9: Matrix metalloproteinase 2, 9; MPO: Myeloperoxidase; NF-кB: nuclear factor κB; PAI-1: Plasminogen activator inhibitor-1; PPAR-γ: Peroxisome proliferator-activated receptor γ; PWV: Pulse wave velocity; S100B: S100 calcium-binding protein B; s.c.: Subcutaneously; SBF: Forearm skin blood flow; SBP: Systolic blood pressure; SHR: Spontaneously hypertensive rat; SOD: Superoxide dismutase; SV: Systolic volume; TNF-α: Tumour necrosis factor α; TPR: Total peripheral resistance; TRPV1: Transient receptor potential vanilloid subfamily member 4; VCAM-1: Vascular cell adhesion protein 1; VEGF: Vascular endothelial growth factor; vWF: von Willebrand factor; ZDF: Zucker Diabetic Fatty rat.

## 5. Effects of Abnormal-Cannabidiol on the Cardiovascular System

**Figure 4:**
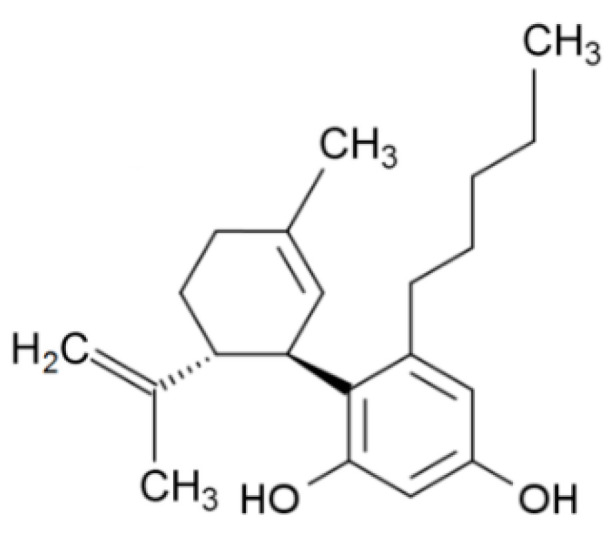
Chemical structure of abnormal-cannabidiol.

Abnormal-cannabidiol (chemical structure Figure 4) is a synthetic regioisomer of cannabidiol which did not exhibit overt behavioural effects in preliminary studies [51,148,149]. Abn-CBD produces hypotension in anaesthetised dogs [148] and mice [51,150] after systemic administration, and vasodilation in isolated mice and rat mesenteric bed [51], rat mesenteric arteries [150,151,152], and rat [153], rabbit [154] and human pulmonary arteries [155]. These effects are not dependent on cannabinoid receptors. Vasorelaxant action of Abn-CBD occurs, hypothetically, via an unidentified endothelial receptor called Abn-CBD receptor or endothelial cannabinoid receptor and is inhibited by CBD and O-1918, the putative antagonists of this receptor [51,71,73]. However, based on current knowledge, the existence of specific endothelial receptor for Abn-CBD is rather controversial. Abn-CBD is an agonist of GPR18, and O-1918 and CBD serve as an antagonist or partial agonist/antagonist of this receptor, respectively [73,156]. Therefore, some authors suggest that the proposed endothelial cannabinoid receptor may be GPR18. However, not all experimental observations confirm it explicitly [73]. In addition, GPR18-independent activation of high-conductance Ca^2+^-activated K^+^ (BK_Ca_) channels might contribute to vasodilatory action of Abn-CBD [157]. Abn-CBD can also lower blood pressure after intra-RVLM administration via GPR18 activation which leads to sympathoinhibition [158]. Moreover, this compound is an agonist of GPR55, however this receptor does not mediate vasorelaxant response to Abn-CBD [150].

Chronically administered Abn-CBD had beneficial effects on the cardiovascular system in rats by lowering the mean blood pressure, increasing plasma and heart adiponectin concentration, increasing the availability of NO in vessels, improving the left ventricular function or decreasing the reactive oxygen species in the heart [159]. Abn-CBD also showed cardioprotective effects in rats with streptozocin-induced diabetes. It alleviated cardiac dysfunction (but not cardiac hypertrophy), vagal dominance, myocardial oxidative stress, and reduced cardiac and/or circulating NO and adiponectin levels in diabetic rats [160]. All these beneficial effects were abrogated by O-1918 indicating the role of GPR18 in protective action of Abn-CBD in the cardiovascular system [159,160]. Cardioprotection observed in diabetic rats was not accompanied with improvement in glycaemic control—Abn-CBD did not influence blood glucose and insulin levels [160]. However, in another studies this compound exhibit antidiabetic potential through GPR55 activation [161,162,163,164]. Abn-CBD also possesses neuroprotective properties, because it reduced the infarct volume as potently as did CBD in mice model of stroke [53]. In summary, both the CBD and its synthetic analog Abn-CBD has vasodilatory properties (at the same time CBD inhibits the vasorelaxant effect of Abn-CBD) and show potential beneficial effects in the cardiovascular system, however via different (even partly opposed) mechanisms. This apparent contradiction could be explained by the complex and multitarget mechanism of CBD action, e.g., (1) the vasodilatory effect of CBD occurs via direct or indirect activation of different receptors (depending on vascular bed) including CB_1_, TRPV1, EP_4_, IP and PPAR-γ [48,56,106]; (2) CBD may act both as an antagonist and a partial agonist of GPR18 [156]; and (3) CBD directly antagonizes GPR55 [11] but it can potentially activate this receptor indirectly via inhibition of endocannabinoid degradation [27].

## 6. Conclusions

**Figure 5:**
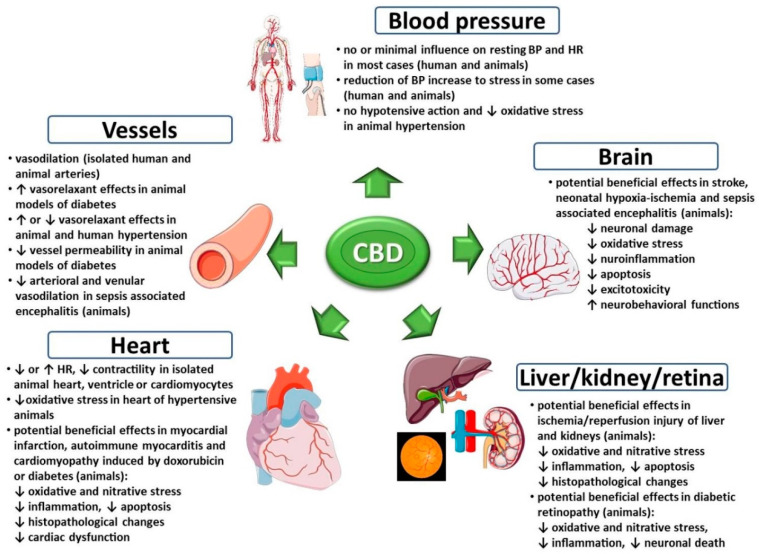
Effects of cannabidiol (CBD) on the cardiovascular system under physiological and pathological conditions [7,15,23,24,47,48,49,50,51,52,53,54,55,56,57,74,75,76,77,78,79,80,81,82,83,84,85,86,87,88,89,90,91,92,93,94,95,96,97,98,99,100,101,102,103,104,105,106,107,108,109,110,111,112,113,114,115,116,117,118,119,120,121,122,123,124,125,126,127,128,129,130,131,132,133,134,135,136,137,138,139,140,141,142,143,144,145,146,147,148]. Abbreviations: BP: blood pressure, HR: heart rate; this figure was prepared using a template on the Servier Medical Art website.

This article has reviewed the effects of cannabidiol, a non-intoxicating cannabis component with a wide therapeutic potential and good safety profile, on the cardiovascular system under physiological and pathological conditions (summarized in Figure 5). CBD might affect the cardiovascular system via different direct and indirect mechanisms. A detailed determination of CBD impact on the cardiovascular system is important considering the still-increased usage of this compound for therapeutic (including self-medication) or recreational purposes. However, with a few exceptions, the effect of CBD on the cardiovascular system under physiological conditions appears to be negligible, which confirms a good safety profile of this cannabinoid. On the other hand, potential CBD application for the treatment of cardiovascular disorders is considered. In experimental pathological conditions, such as hypertension, heart diseases, stroke, neonatal hypoxia-ischemia, diabetes or hepatic and renal ischemia/reperfusion injury, the protective effect of CBD associated with its anti-inflammatory, antioxidant, antiapoptotic, vasculoprotective, cardioprotective or neuroprotective effects is often revealed. Despite its vasodilatory properties, CBD has not been demonstrated to exhibit hypotensive action in animal models of hypertension. However, this compound might lower stress-induced increases in blood pressure in both humans and animals. Nevertheless, it should be emphasized that almost no clinical research has been done with CBD in diseases of the cardiovascular system and, hence, its therapeutic potential is not translated into clinical practice. Further studies, especially clinical investigations, are warranted to recommend the use of CBD in the treatment of cardiovascular disorders.

## Figures and Tables

**Table 1:** Comparison of the main cannabidiol and Δ^9^-tetrahydrocannabinol properties [4,6,8,9,10,11,12,13,14,15,16,17,18,19,20,21,22,23,24,25,26,27,28,29,30,31,32,33].

	Cannabidiol (CBD)	Δ^9^-Tetrahydrocannabinol (THC)
Structure and IUPAC name	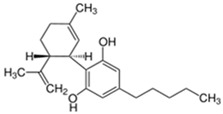 2-[(1R,6R)-3-Methyl-6-prop-1-en-2-ylcyclohex-2-en-1-yl]-5-pentylbenzene-1,3-diol	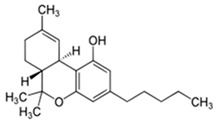 (6aR,10aR)-6,6,9-Trimethyl-3-pentyl-6a,7,8,10a-tetrahydrobenzo[c]chromen-1-ol
Psychoactive properties	Psychoactive ^1^ but non-intoxicating; does not produce cannabinoid tetrad ^2^	Psychoactive and intoxicating (‘high’, euphoria, sensations of pleasure and relaxation, psychomotor and cognition impairment); produces cannabinoid tetrad ^2^
Potential therapeutic properties ^3^	Anti-inflammatory, antioxidant, immunomodulatory, neuroprotective, anticonvulsant, anxiolytic, antipsychotic, antidepressant, procognitive, antiarthritic, analgesic, antiemetic, anticancer, cardioprotective, vasodilatory	Analgesic, antispastic, anti-inflammatory, appetite stimulant, antiemetic, neuroprotective, anxiolytic, antiasthmatic, antiglaucomatous, anticancer
Pharmaceutical products	Dried female cannabis flowers (‘medical marijuana’) and their derivatives (oil, granulate) with different THC:CBD ratios (e.g., Bedrocan^®^ products) nabiximols (Sativex^®^)—cannabis extract containing CBD and THC in a ~1:1 ratio	
	Cannabis-derived CBD (Epidiolex^®^)	Dronabinol (Marinol^®^, Syndros^®^)—synthetic THC Nabilon (Cesamet^®^, Canemes^®^)—synthetic THC analogue
Hypothesized mechanism of action	Affinity for cannabinoid receptors CB_1_ (K_i_ = 4350 to >10,000 nM) CB_2_ (K_i_ = 2399 to >10,000 nM) Antagonist of CB_1_/CB_2_ receptor agonists, negative allosteric modulator of CB_1_ and inverse agonist of CB_2_	Affinity for cannabinoid receptors CB_1_ (K_i_ = 5.05–80.03 nM) CB_2_ (K_i_ = 3.13–75.3 nM) Partial agonist of CB_1_ and CB_2_
	Indirect cannabimimetics: ↑AEA, 2-AG (inhibits FAAH and AEA uptake by binding to EMT and FABP-3, -5, -7)	Indirect cannabimimetics: ↑AEA (inhibits AEA re-uptake by binding to FABP-3, -5, -7)
	(+) TRPA1, TRPV1–4, PPAR-γ, 5-HT_1A_, 5-HT_2A_, α1-, α1β-, α3-GlyR,μ-, δ-OR, GABA_A_ (–) GPR55, GPR3, GPR6, GPR12, GPR18 ^4^,Abn-CBD receptor, TRPM8, 5-HT_3_, α_1_-AR, D_2_ Affects uptake/metabolism of adenosine, glutamate, serotonin, dopamine, γ-aminobutyric acid, noradrenaline, tryptophan, arachidonic acid	(+) GPR55, GPR18, PPAR-γ, TRPA1, TRPV2, 5-HT_2A_, α1- and α1β1-GlyR (–) 5-HT_3_, μ- and δ-OR, TRPM8 Affects uptake/metabolism of adenosine, serotonin, γ-aminobutyric acid, dopamine, noradrenaline, arachidonic acid
Influence on cardiovascular system (physiological conditions)	No or slight influence on BP and HR in human (usually) No or slight influence on BP and HR in animals (usually) Vasodilation of isolated vessels	↑ HR (significant) and ↑ or ↓ BP in human ↓ HR (usually), and ↓ or ↑ or biphasic changes in BP in animals Vasodilation or vasoconstriction of isolated vessels

^1^ CBD is considered psychoactive due to its anti-anxiety, antipsychotic and antidepressant effects; ^2^ cannabinoid tetrad is characterized by hypolocomotion, hypothermia, catalepsy and antinociception induced by THC and other psychoactive cannabinoids (agonists of CB_1_) in mice; ^3^ based on preclinical and clinical studies; registered indications (USA and/or EU) include only spasticity in multiple sclerosis (Sativex^®^), drug-resistant epilepsy—Dravet syndrome and Lennox-Gastaut syndrome (Epidiolex^®^), chemotherapy-induced nausea and vomiting (Marinol^®^, Syndros^®^, Cesamet^®^, Canemes^®^), and AIDS-associated anorexia (Marinol^®^, Syndros^®^); ^4^ CBD is a low efficacy partial agonist of GPR18 and antagonizes THC effects (CBD acts as an antagonist); ↑/↓—increase/decrease; (+)—agonist or positive allosteric modulator; (–)—antagonist, inverse agonist or negative allosteric modulator; abbreviations: 2-AG: 2-arachidonoylglycerol; 5-HT_1A, 2A, 3_: serotonin receptors type 1A, 2A, 3; Abn-CBD: abnormal-cannabidiol; AEA: anandamide; BP: blood pressure; CB_1, 2_: cannabinoid receptor type 1, 2; D_2_: dopamine receptor type 2; EMT: endocannabinoid membrane transporter; FAAH: fatty acid amide hydrolase; FABP-3,-5,-7: fatty acid binding protein 3, 5, 7; GABA_A_: γ-aminobutyric acid receptor type A; GPR3, 6, 12, 18, 55: G-protein coupled receptor 3, 6, 12, 18, 55; HR: heart rate; PPAR-γ: peroxisome proliferator-activated receptor γ; TRPA1: tansient receptor potential ankyrin subfamily member 1; TRPM8: transient receptor potential melastatin subfamily member 8; TRPV1-4: transient receptor potential vanilloid subfamily members 1-4; α1-, α1β-, α3-GlyR: α1, α1β-, α3-glycine receptor; α_1_-AR: α_1_-adrenergic receptor; δ-, μ-OR: δ-, μ-opioid receptor.

**Table 2:** In vivo effects of cannabidiol (CBD) in cardiovascular system under physiological ^1^ conditions.

Species	Anaesthesia	Route	Dose	Effects ^2^	References
Single administration					
human	-	p.o.	320 µg/kg	↔ HR	[75]
human	-	p.o.	1 mg/kg	↔ HR	[77]
human	-	p.o.	100; 600; 1200 mg	↔ DBP, SBP, HR	[78]
human	-	p.o.	300 mg	↔ SBP, HR	[80]
human	-	p.o.	400 mg	↑ CBF (regional)	[101]
human ^3^	-	s.l.	20; 40 mg	↑ SBP ↔ DBP, HR	[98]
human	-	p.o.	600 mg	↔ SBP, DBP, HR	[83]
human	-	p.o.	600 mg	↔ BP, HR	[84]
human	-	p.o.	600 mg	↔ BP, HR	[85]
human	-	p.o.	600 mg	↔ BP, HR	[86]
human	-	p.o.	600 mg	↔ DBP, SBP, HR	[88]
human	-	p.o.	600 mg	↔ BP, HR	[89]
human	-	p.o.	600 mg	↔ DBP, SBP, HR	[91]
human	-	p.o.	200; 400; 800 mg	↔ DBP, SBP, HR	[93]
human	-	p.o.	600 mg	↓ SBP, DBP, MBP, SV, TPR, SBF ↑ HR ↔ CO, EJT	[99]
human	-	p.o.	45; 90 mg	↔ SBP, DBP, MBP, HR, CBF	[94]
			45; 90 mg TurboCBD^TM 4^	↔ SBP, HR ↓ DBP, MBP ↑ CBF	
human		inhalation (vaporisation)	400 mg	↔ HR, SBP, DBP (↑ DBP in frequent cannabis users)	[7]
human ^5^	-	inhalation (smoking)	1/2 of cigarette containing ~800 mg of cannabis (0.4% THC/10.4% CBD)	↔ SBP, DBP, HR	[95]
human	-	p.o.	600 mg	↓ MBP ↔ SBP, DBP, HR, CO, SV, EJT, TPR	[97]
dog	pentobarbital	i.v.	0.5; 1 mg/kg	↑ MBP, HR	[102]
rabbit	-	i.v.	25 mg/kg	↓ HR	[103]
rat	-	i.p.	10 mg/kg	↔ MBP, HR	[82]
rat	-	i.p.	1; 10; 20 mg/kg	↔ MBP, HR	[55]
rat	-	i.p.	10 mg/kg	↑ (slight) SBP, DBP, HR	[52]
rat	urethan	i.v.	1 mg/kg	↔ BP, HR	[74]
rat	urethan	i.v. (rapid)	3; 10; 30 mg/kg	↓ SBP, DBP, HR (Bezold-Jarisch reflex induced via TRPV1) ↓ Bezold-Jarisch reflex induced by 5-HT_3_ (but not TRPV1) activation	[55]
rat ^6^	urethane	i.v.	1; 3; 30 mg/kg	↑ SBP, HR ↓ DBP	[55]
rat	pentobarbital	i.a. or i.v.	1-2000 µg	↔ MBP	[81]
rat	pentobarbital	i.v.	10; 50 µg/kg	↓ MBP ↔ HR	[105]
rat	pentobarbital	i.v.	50 µg/kg	↓ MBP ↔ HR	[104]
rat	thiopental	i.v.	50 µg/kg	↔ MBP, HR	[49]
rat	-	i.c.	15; 30; 60 nmol	↔ MBP, HR	[87]
rat	-	into BNST	15; 30; 60 nmol	↔ MBP, HR	[90]
rat	-	into BNST	15; 30; 60 nmol	↔ MBP, HR	[92]
rat	-	into BNST	60 nmol	↔ MBP, HR ↑ reflex bradycardiac response to BP increase (effect is dependent on 5-HT_1A_) ↔ reflex tachycardiac response to BP decrease	[47]
mouse	ketamine + xylazine	i.v.	50 µg/kg	↓ MBP ↔ HR	[104]
**Chronic administration**					
human	-	p.o.	3 mg/kg for 30 days	↔ HR, ECG	[76]
human ^7^	-	p.o.	200-300 mg for 4,5 months	↔ HR, ECG	[76]
human	-	p.o.	1200 mg for 20 days	↔ DBP, SBP, HR	[78]
human ^8^	-	p.o.	increasing doses 100-600 mg for 6 weeks	↓ BP	[100]
human ^9^	-	p.o.	10 mg/kg/day over 6 weeks	↔ MBP, HR	[79]
human ^10^	-	p.o.	800 ^11^ mg for 4 weeks	↔ SBP, DBP, HR	[15]
human	-	p.o.	600 mg for 7 days	↔ SBP, DBP, MBP, HR ↑ PWV, FMD	[97]
rat	-	i.p.	10 mg/kg for 14 days	↔ SBP, DBP, HR ^11,12^↑ oxidative stress markers in plasma (MDA ^11^, 4-HHE ^11,12^, 4-HNE ^11^) and in heart (MDA^11^, 4-HHE ^11^, 4-HNE ^11^)	[96]

^1^ concerning only cardiovascular system; ^2^ effects observed with at least one of the tested doses; ^3^ patients with glaucoma; ^4^ TurboCBD^TM^ is a patented capsule formulation of CBD increasing its bioavailability (45 or 90 mg CBD, 600 mg American ginseng, 240 mg *Ginkgo biloba*, 150 mg organic hemp oil); ^5^ patients with obsessive-compulsive disorder; ^6^ pithed and vagotomised rat; ^7^ patients with epilepsy; ^8^ patients with dystonic movement disorders; ^9^ patients with Huntington’s disease; ^10^ patients with schizophrenia; ^11^ treatment started with 200 mg/day and increased stepwise by 200 mg/day to a daily dose of 800 mg/day (200 mg four times a day) within the first week (in some patients treatment was reduced to 600 mg/day after two weeks due to side effects); ^11^ normotensive control rats for SHR (Wistar-Kyoto rats); ^12^ normotensive control rats for DOCA-salt rats; ↑/↓/↔—increase/decrease/no change; abbreviations: 4-HHE: 4-Hydroxyhexenal; 4-HNE: 4-Hydroxynonenal; 5-HT_1A,3_: Serotonin receptors type 1A, 3; BNST: Bed nucleus of the stria terminalis; BP: Blood pressure; CO: Cardiac output; DBP: Diastolic blood pressure; ECG: Electrocardiogram; EJT: Left ventricular ejection time; FMD: Flow mediation dilatation; HR: Heart rate; i.a.: Intra-arterially; i.c.: Intracisternally; i.p.: Intraperitoneally; i.v.: Intravenously; MBP: Mean blood pressure; MDA: Malondialdehyde; p.o.: Per os, orally; PWV: Pulse wave velocity; s.l.: Sublingually; SBF: Forearm skin blood flow; SBP: systolic blood pressure; SV: systolic volume; THC –Δ^9^–Tetrahydrocannabinol; TPR: Total peripheral resistance; TRPV1: Transient receptor potential vanilloid subfamily member 4.

## Abbreviations

2-AG	2-Arachidonoylglycerol
5-, 15-LOX	5-, 15-Lipooxygenase
5-HT_1A/2A/3_	Serotonin receptors type 1A,2A, 3
7-COOH-, 7-OH-CBD	7-Carboxy-, 7-hydroxycannabidiol
A_1/2_	Adenosine receptor type 1, 2
Abn-CBD	Abnormal-cannabidiol
AEA	Anandamide
Akt	Protein kinase B
BNST	Bed nucleus of the stria terminalis
BP	Blood pressure
CB_1, 2_	Cannabinoid receptor type 1, 2
CBC	Cannabichromene
CBCA	Cannabichromenic acid
CBD	Cannabidiol
CBF	Cerebral blood flow
CBGA	Cannabigerolic acid
COX-1, -2	Cyclooxygenase 1, 2
CREB	cAMP response element-binding protein
CYP	Cytochrome P450
D_2_	Dopamine receptor type 2
DBP	Diastolic blood pressure
DMAPP	Dimethylallyl diphosphate
DOCA-salt	Deoxycorticosterone acetate-salt (model of hypertension)
EMT	Endocannabinoid membrane transporter
EP_1, 4_	Prostaglandin E receptor 1, 4
ERK1/2	Extracellular signal-regulated kinase 1/2
FAAH	Fatty acid amide hydrolase
FABP-3, -5, -7	Fatty acid binding protein 3, 5, 7
GABA_A_	γ-Aminobutyric acid receptor type A
GPP	Geranyl diphosphate
GPR3, 6, 12, 18, 55	G-protein coupled receptor 3, 6, 12, 18, 55
HIE	Hypoxic-ischemic encephalopathy
HR	Heart rate
i.p.	Intraperitoneally
i.v.	Intravenously
ICAM-1	Intercellular adhesion molecule 1
INR	International normalized ratio
IP	Prostacyclin receptor
IPP	Isopentenyl diphosphate
JNK	c-Jun N-terminal kinase
LPS	Lipopolysaccharide
MAPK	Mitogen-activated protein kinases
MBP	Mean blood pressure
MCA	Middle cerebral artery
MEP	2-Methylerythritol 4-phosphate
MEV	Mevalonic acid
NF-кB	Nuclear factor κB
OA	Olivetoleic acid
p.o.	Per os, orally
p70S6K	Ribosomal protein S6 kinase
PGE	Prostaglandin E
PLA_2_	Phospholipase A_2_
PPAR-γ	Peroxisome proliferator-activated receptor γ
RVLM	Rostral ventrolateral medulla
SBP	Systolic blood pressure
SHR	Spontaneously hypertensive rat
STAT5	Signal transducer and activator of transcription 5
THC	Δ^9^-Tetrahydrocannabinol
THCA	Δ^9^-Tetrahydrocannabinolic acid
TNF-α	Tumour necrosis factor α
TP	Thromboxane receptor
TRP	Transient receptor potential
TRPA1	Transient receptor potential ankyrin subfamily member 1
TRPM8	Transient receptor potential melastatin subfamily member 8
TRPV1-4	Transient receptor potential vanilloid subfamily members 1-4
VCAM-1	Vascular cell adhesion protein 1
VEGF	Vascular endothelial growth factor
ZDF	Zucker Diabetic Fatty rat
α1-, α1β-, α3-GlyR	α1, α1β-, α3-Glycine receptor
α_1_-AR	α_1_-Adrenergic receptor
δ-, μ-OR	δ-, μ-Opioid receptor
